# Effects of Photobiomodulation Therapy on Pain and Healing of Episiotomies and Grade 2 and 3 Perineal Lacerations After Vaginal Delivery: A Prospective Observational Cohort Study

**DOI:** 10.3390/medsci14010125

**Published:** 2026-03-06

**Authors:** Luir Jose Ruaro Filho, Mônica Vieira Barcellos, Flavia Bezerra Provazi Pesci, Larissa Camargo e Silva, Juliana Carla Eleutério dos Santos, Alexia Jacobs, Ana Flávia Araújo Litwinczuk, Maria Fernanda Setúbal Destro Rodrigues, Lara Jansiski Motta, Carlos Souto dos Santos Filho, Rebeca Boltes Cecatto

**Affiliations:** 1 Biophotonics-Medicine Post-Graduate Program, Universidade Nove de Julho—UNINOVE, 249 Av Vergueiro, Liberdade, São Paulo 01504-001, SP, Brazil; luir.ruaro@bol.com.br (L.J.R.F.); anaflavia70@uni9.edu.br (A.F.A.L.); fernandarodrigues@uni9.pro.br (M.F.S.D.R.); ljm.larajmotta@gmail.com (L.J.M.); 2 Hospital Universitário Materno-Infantil, Universidade Estadual de Ponta Grossa, Ponta Grossa 84010-330, PR, Brazil; barcellos_monica@hotmail.com (M.V.B.); flaviapesci2@gmail.com (F.B.P.P.); laricamargo008@gmail.com (L.C.e.S.); jucarlasantos@hotmail.com (J.C.E.d.S.); alexiajacobsfisioterapeuta@gmail.com (A.J.); 3 Graduate Program in Statistics, Institute of Mathematics, Statistics and Scientific Computing, State University of Campinas—UNICAMP, Rua Sérgio Buarque de Holanda, 651, Campinas 13083-859, SP, Brazil; c300761@dac.unicamp.br; 4 Rehabilitation Service of the Instituto de Reabilitação Lucy Montoro, Faculdade de Medicina FMUSP, Universidade de São Paulo, São Paulo 05716-150, SP, Brazil

**Keywords:** implementation science, episiotomy, pain, healing, photobiomodulation, vaginal delivery, perineal lacerations

## Abstract

Tissue trauma related to operative vaginal delivery or the use of episiotomy has been associated with complications such as pain and infection, and successful management is a key factor in promoting a faster and better recovery after delivery. Strategies that promote analgesia and tissue regeneration are especially relevant in obstetric care. **Background/Objectives**: To evaluate the effects of photobiomodulation (PBM) on pain and perineal healing in women who underwent episiotomy or sustained second- and third-degree lacerations after vaginal delivery. **Methods**: Observational cohort study. PBM is routinely offered postpartum as an adjuvant to standard hospital treatment daily throughout hospitalization for postpartum women who underwent episiotomy or second- and third-degree lacerations after vaginal delivery, recruited over a prospective 6-month period. The treatment protocol used an 808 nm laser at 100 mW, applied to five perilesional points at 4 J/cm^2^ daily during hospitalization. Propensity score matching (PSM) analysis and two-way ANOVA were used to compare subgroups of patients who agreed to receive adjuvant PBM and those who did not, in addition to conventional treatment. Daily pain was assessed using the numerical pain scale (NPS) before and after PBM and conventional treatment, and healing was evaluated using the REEDA scale. **Results**: Data from 149 PBM-treated women and 34 non-PBM-treated women were analyzed. After PSM matching, a mean difference in NPS scores of 1.6761 (standard error = 0.4379) was observed between the group submitted to two laser sessions and the no-laser group, indicating a statistically significant difference (F = 14.65; Pr = 0.000191). A significant decrease in NPS scores was observed with ANOVA before and after each PBM application (*p* < 0.05), over the three days of follow-up. Regarding the REEDA scale, the preliminary results indicate a trend toward lower scores (better outcomes) in the laser group, an effect that larger studies could confirm. **Conclusions**: PBM was associated with improvements in pain reduction and tissue healing.

## 1. Introduction

Successful pain management in the perioperative phase is a key factor in promoting a faster and better recovery after surgery, surgical procedures, or complications [1]. Multiple studies have developed evidence-based recommendations for pain treatment options and strategies for surgical pain management [2]. In the obstetric context, tissue trauma related to operative vaginal delivery or the use of episiotomy has been associated with complications such as pain and infection [3]. Perineal lacerations, especially second- and third-degree lacerations, are also common and can cause persistent intense pain, as well as complications such as incontinence, fistulas, and sexual dysfunction [4]. Identifying and properly repairing the muscular structures of the perineum are essential for recovery and preventing the sequelae of obstetric tissue trauma. In this scenario, strategies that promote analgesia and tissue regeneration are especially relevant in obstetric care.

Photobiomodulation (PBM), formerly known as low-level laser therapy [5,6], has emerged as a promising and safe approach for pain control and healing stimulation. It uses low-intensity light (laser or LED) to act on chromophores, stimulating ATP production, nitric oxide release, and modulation of inflammatory cytokines [7,8,9]. Clinical studies demonstrate its analgesic and regenerative effects in different tissues, with emphasis on its application in postoperative care and dentistry, without significant side effects [10].

Despite growing interest in PBM for analgesia, the current obstetric evidence remains limited and heterogeneous. Previous studies evaluating PBM after episiotomy or perineal lacerations have generally involved small samples, short follow-up periods, and variability in treatment parameters, such as wavelength, energy dose, timing, and number of sessions, making it difficult to establish standardized clinical recommendations. Although still underexplored in the obstetric field, PBM has shown potential in relieving pain and promoting healing after perineal trauma, incisions, and wounds [11]. Furthermore, there is limited evidence on PBM effectiveness in real-world obstetric settings, where variations in patient characteristics, clinical practices, and healing processes may influence outcomes. Important uncertainties, therefore, remain regarding optimal treatment protocols and the magnitude of PBM benefits on pain reduction and tissue repair in routine care. In this context, a real-world cohort design allows for the evaluation of PBM under everyday clinical conditions, providing pragmatic evidence on its effectiveness and safety and supporting its potential incorporation into obstetric practice.

Based on these data, this study was developed to evaluate the effects of PBM on pain and perineal healing in women undergoing episiotomy or second- and third-degree lacerations after vaginal delivery using the numerical pain scale (NPS) and the REEDA scale as assessment instruments. The hypothesis of this study is that PBM contributes significantly to improving these outcomes.

## 2. Materials and Methods

### 2.1. Study Design and Setting

This prospective observational cohort study was conducted over six months. At the obstetrics facility of the University hospital, the institution’s physiotherapy team routinely offers conventional therapies and perineal laser therapy for pain control to all women who undergo episiotomy or sustain second-degree or higher perineal tears after childbirth. The vast majority of women accept the treatment and report symptom improvement after laser application. As the laser application protocol described in this study is already part of the standard care provided by the hospital’s physiotherapy team, this observational study did not interfere in any way with the therapeutic care women received during hospitalization and was conducted as a prospective observational cohort. All women being followed up at the service during the six-month period of study recruitment were assessed regarding the study inclusion criteria and invited to participate. This protocol complies with the STROBE guidelines and its RECORD statement extension for the observational description of already implemented clinical care protocols [12,13].

### 2.2. Ethical Aspects of Research

The participation of each patient was conditioned on the signing of the Free and Informed Consent Form previously approved by the Ethics and Research Committee of the University on 8 August 2024, under CAAE 81847124.6.0000.0105/number 6,993,678. All participants were maintained under medical routine follow-up and health program at the hospital. All data analyzed and published are anonymized, maintaining confidentiality regarding the identification of participants.

### 2.3. Participants

Inclusion criteria were postpartum women aged ≥18 years who sustained episiotomy or second-/third-degree perineal lacerations following vaginal delivery. Exclusion criteria included gestational age < 37 weeks, systemic illness affecting healing, peripartum complications, such as postpartum hemorrhage, multiple pregnancy (twins), fetal malformations, or fetal death identified during prenatal care or delivery, and patients with severe complications, such as major postpartum hemorrhage or HELLP syndrome during delivery.

### 2.4. Routine Therapeutic Procedures

In the routine health care of the hospital, all women undergoing episiotomy or perineal laceration receive conventional therapies for pain control: analgesics, anti-inflammatories, and ice packs. It is also routine to offer PBM, and it is up to the women to accept or not after guidance from the physiotherapy team. For women who spontaneously accept, PBM is applied between two and 18 h after delivery and once a day throughout the hospital stay by the physiotherapy team. Contraindications include the presence of cancer, photosensitivity, undiagnosed lesions, and use of photosensitizing substances. The equipment used is the MMO Recover^®^ (MMOptics, São Carlos, Brazil; 808 nm, 100 mW, 4 J/cm^2^), with application at five skin points of the cutaneous perineum close to the lesion. All parameters used are presented in Table 1.

### 2.5. Outcomes

Data regarding the chosen outcomes were collected from the medical records of the included participants and correspond to the daily measurements carried out routinely during the three-day hospitalization period, respecting the standardized moment for discharge after vaginal birth decided by the medical care team.

#### 2.5.1. Primary Outcome

Pain is routinely assessed by physiotherapists daily using the numerical pain scale (NPS) [14]. Women reported their pain perception before the treatment (conventional and/or PBM sessions) and also after each PBM session for participants who used PBM, with zero representing “no pain” and 10 representing “worst possible pain”. The NPS is a commonly used instrument for pain measurement. It is quick and easy to administer and easily understood by patients, making it an appropriate method for estimating pain intensity [15]. The NPS is printed on the patient assessment form, and participants are instructed to mark a point on a 10 cm line indicating their pain intensity before and after the procedure. It consists of a straight line with the marking “no pain” at one end and “worst possible pain” at the other.

#### 2.5.2. Secondary Outcome

Perineal healing was assessed daily by the principal investigator (PI) using the REEDA scale, observing local hyperemia, edema, ecchymosis, secretion, and wound coaptation, with a total score of zero to 15 [16]. The REEDA scale is an instrument for assessing perineal healing developed by Davidson [17]. It includes five items related to the healing process: redness, edema, ecchymosis, discharge, and approximation of the wound edges. This scale can be used to evaluate all types of postpartum perineal trauma. The REEDA score ranges from 0 (indicating complete healing and closure of the lesion) to a maximum score of 15, which corresponds to the worst perineal healing with failure of approximation of the skin, subcutaneous tissue, and musculature.

#### 2.5.3. Outcomes Analysis

In our hospital, PBM is routinely offered to all patients with episiotomies and perineal lacerations after vaginal delivery. The decision to accept the treatment is made by the patient. For all analyses regarding outcomes, comparisons were made between subgroups of participants who chose to accept adjuvant laser associated with conventional treatment and the subgroup of participants who chose only conventional treatment. Since participants have the right to refuse or accept the laser according to their own wishes, they voluntarily transitioned between subgroups during hospitalization primarily based on their pain levels and perceived need for additional analgesic treatment. In this way, the cohort composition changed daily due to participant migration between laser options. This situation posed a methodological challenge for analysis due to changes in treatment groups, resulting in multiple possible analytical perspectives for the subgroups formed. To address this issue, participants were pre-specified and classified according to their treatment choice on the first evaluation day (day 1), which reflects the initial clinical decision context and minimizes bias related to changes occurring after treatment initiation. This approach was chosen to approximate an intention-to-treat framework, preserving baseline comparability and allowing evaluation of PBM effectiveness as implemented in routine clinical practice.

In addition, secondary exploratory analyses considered the cumulative number of PBM sessions received to assess potential dose–response effects, acknowledging variability in exposure due to hospital discharge timing and clinical evolution. The primary objective of the study was to evaluate PBM effectiveness under real-world conditions rather than efficacy under controlled exposure; therefore, classification based on the initial treatment decision was considered the most appropriate approach to reflect pragmatic clinical use.

The outcomes were analyzed by comparing pre-treatment NPS and REEDA scores between the first, second, and third day, and also between subgroups (laser or no laser) by analyzing daily measurements described. A comparison between before and after PBM moments for NPS was also made within the subgroup of participants who accepted PBM. For comparisons about measurements taken across the three days of hospitalization, we also chose to compare REEDA and NPS scores among three other subgroups (that emerged from participants’ spontaneous choice): participants who chose not to undergo the laser on any day; participants who underwent only one laser session throughout their three days of hospitalization; and women who underwent two laser sessions throughout three days of hospitalization.

The NPS and the REEDA scale are appropriate tools for evaluating different dimensions of postoperative recovery because they allow simple and standardized measurement of key aspects of recovery, such as pain and tissue healing. The NPS acts as a sensitive instrument for assessing the intensity of pain perceived by the patient, enabling monitoring of clinical evolution over time and evaluation of the response to the instituted treatment, as well as facilitating comparisons across different time points and groups. The REEDA scale complements this assessment by providing a structured clinical evaluation of perineal healing, including inflammatory signs and wound integrity, allowing early identification of changes in the tissue repair process and systematic monitoring of healing progression.

### 2.6. Statistical Analysis

The data were tabulated in Excel, and descriptive statistics (mean, standard deviation) were calculated. Normality was assessed with the Kolmogorov–Smirnov test. Continuous variables are expressed as mean ± standard deviation (SD). The mean was calculated as the arithmetic average of the observations, and the SD was calculated as the square root of the sample variance. Categorical variables are presented as absolute frequencies (*n*) and proportions when appropriate. Student’s *t*-test and the chi-square test were used for continuous and categorical variables of sample characteristics, respectively. The primary outcome of this study was to analyze the between-group differences regarding the use of laser therapy for pain management and perineal healing. For this, an initial exploratory comparison of outcomes used repeated-measures ANOVA in SPSS 24.0 (significance level: 5%). Due to the observational design and sample characteristics, the subgroups (laser vs. no laser) had different sample sizes and measurement numbers across the three-day follow-up. To increase reliability, a propensity score matching (PSM) analysis was performed to control for bias and balance covariates. The PSM is a very robust, rigorous, and more reliable test in situations where the sample size is small or the groups being compared are unbalanced. PSM offers advantages over traditional ANOVA, particularly in small samples where ANOVA assumptions are rarely met, including the flexibility to use different probability distributions and covariate analysis. After matching, treatment effects were estimated using Welch’s *t*-test and ordinary least squares regression, validated with Monte Carlo simulations. The PSM was conducted in R (version 4.4.3) using R Studio, with the code written by C.S.d.S.F. All propensity scores matching comparisons, along with comprehensive documentation of the PSM analytical process (including all calculations, tables, and graphs), are available in Supplementary File S1.

### 2.7. Patient and Public Involvement

Patients or the public were not involved in the design, conduct, reporting, or dissemination plans of our research.

## 3. Results

**Figure 1:**
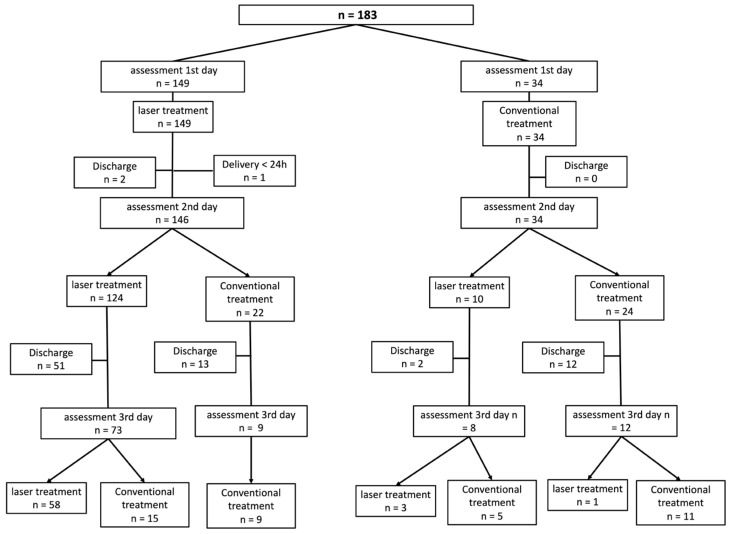
Recruitment, intervention, and evaluation flowchart, which refers to participants’ choices, treatments, and discharges during the entire study follow-up period.

This study evaluated one hundred and ninety-eight women over a six-month recruitment period. Fifteen women were excluded prior to the follow-up phase: one for refusing to participate, eight for being under 18 years of age, and six for having a gestational age of less than 37 weeks at delivery. Following the application of exclusion criteria, 183 participants were enrolled, evaluated, and followed up. As an observational study, the women continued their routine medical follow-up with the hospital team. Over the three-day follow-up period, some participants were discharged from the hospital due to clinical improvement before completing all three scheduled study assessments (conducted on the first, second, or third day of hospitalization). Furthermore, as participants had the right to accept or refuse laser therapy based on their personal preference, they voluntarily transitioned between subgroups during hospitalization. This decision was primarily influenced by their pain levels and perceived need for additional analgesic treatment. For these reasons—participant migration between study arms and the occurrence of early discharges—the cohort composition changed daily. Figure 1 presents the flowchart for recruitment, inclusion, the therapeutic period, and the assessments.

Throughout hospitalization, all women in both subgroups received the same standard care regimen protocol, which consisted of analgesics administered four times daily and anti-inflammatory drugs three times daily. No participant required supplemental analgesia beyond the prescribed standard regimen or the offered PBM (photobiomodulation) protocol. Furthermore, no adverse events were documented during the study period or throughout monitoring from labour until hospital discharge. Table 2 characterizes the sample of 183 participants, initially divided into two subgroups.

**Figure 2:**
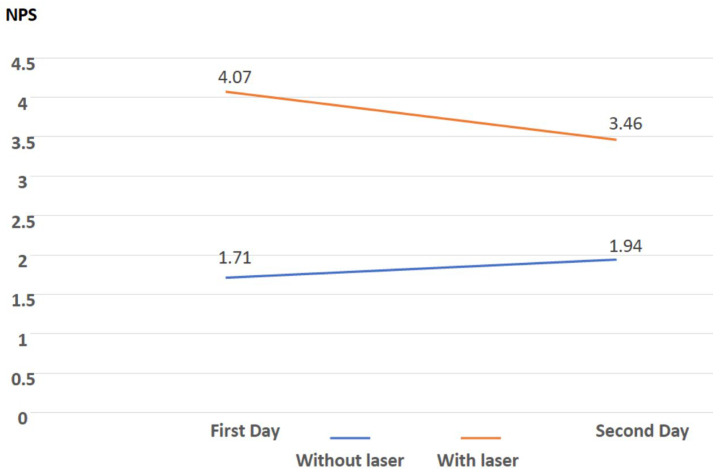
Mean NPS values (*y*-axis) on the first and second day of assessment (*x*-axis), 24 h after the first assessment on subgroups of patients (PBM and no laser option on the first day).

The PBM subgroup comprised participants who chose to undergo laser treatment on the first evaluation day, and the conventional therapy subgroup consisted of participants who opted not to receive laser therapy on the first day. A statistically significant difference was observed between these subgroups for Apgar scores at the first and fifth minutes, with higher (i.e., better) scores recorded among women who declined laser therapy on the first day. This subgroup also presented with smaller perineal tears upon initial evaluation. Among the 149 participants who underwent laser treatment on the first day, three were discharged from the hospital as they were clinically stable, without pain or complications. An initial exploratory repeated-measures ANOVA comparison of the 146 remaining participants showed that the mean NPS decreased significantly from 4.07 to 3.46 by the second baseline assessment. These differences were statistically significant within the PBM group across days (within-person pre/post PBM group). In contrast, the group receiving conventional treatment alone exhibited an increase in scores from 1.71 to 1.94 over the same period, indicating a worsened pain perception, albeit without a statistically significant difference (within-person pre/post conventional treatment group). The evolution of NPS scores between the first and second assessment days is shown in Figure 2 and Table 3.

In the intra-subgroups comparison of NPS values according to the number of laser sessions received over the three days, a repeated-measures ANOVA found significant differences for women who underwent one session (*n* = 17, *p* = 0.016) and for those who underwent two sessions (*n* = 73, *p* < 0.0001) across the three-day assessment period. No significant difference was found for the subgroup receiving zero sessions (*n* = 12). These results are detailed in Table 4 and Supplementary Material Figure S1.

Furthermore, the PSM matching found a mean difference in NPS scores of 1.6761 (standard error = 0.4379) observed between the group that received two laser sessions and the group that received no laser treatment throughout the three-day evaluation period—a between-group difference (Table 5). The result indicates a statistically significant difference between these subgroups (F = 14.65; Pr = 0.000191).

Regarding laser therapy sessions and intragroup analysis (within-person pre/post PBM group), Table 6 shows the comparison of mean NPS scores before and after each application. The repeated-measures ANOVA revealed that post-laser pain scores differed significantly from pre-laser scores after each session in the three days (*p* < 0.001).

Unfortunately, the PSM comparison performed between the subgroup that received one laser session and the subgroup that received zero sessions (between-group differences) showed no statistical difference in pain treatment (*p* = 0.144) between a single laser session and conventional therapy (Supplementary Table S1). Similarly, the PSM comparison between subgroups receiving two laser sessions and one session also revealed no statistically significant difference in pain reduction (*p* = 0.306) over the three-day follow-up period (Supplementary Table S2).

Regarding wound healing (REEDA scale), an initial exploratory repeated-measures ANOVA comparison of the participants who underwent laser treatment on the first day showed improvement between days one and two, decreasing from 1.82 to 1.42 (*p* < 0.0001), a within-person pre/post PBM group difference. In contrast, women who did not receive laser on the first day exhibited worsened REEDA scores without a statistical difference between days 1 and 2 (1.26 to 1.35—Table 7).

Moreover, the subgroup receiving no laser session during three days showed stable or slightly worsened scores (1.42, 1.50, 1.50), while the two-session subgroup saw a significant improvement in the REEDA score from 1.92 to 1.12 across three days in terms of within-group and between-day differences (Supplementary Table S3, Figure S2). A PSM analysis of REEDA scale values found a marginal treatment effect favoring the two-session group over the zero-session group (between-group differences). A mean difference in healing scores (REEDA) of −0.203808 (standard error = 0.114048, F = 106.4) was observed between the group with two laser sessions and the no-laser group. This analysis did not confirm that differences reached the predefined significance threshold of 0.05 for REEDA score progression, and it is not sufficient to conclude a statistically significant difference at an alpha of 0.05 (Pr = 0.07604); a statistically significant difference at a relaxed alpha level of 0.1 (10%) favored the laser-treated group (Supplementary Material Table S4). For the treatment effect (two sessions vs. zero sessions), there is a trend toward lower REEDA scores (better outcome) in the laser group (negative coefficient). This result suggests a potential benefit that will probably only be demonstrated in studies with longer post-therapy evaluation times. No other significant differences were found in the other analyses (Supplementary Materials Tables S5 and S6).

## 4. Discussion

The management of perineal pain and the promotion of wound healing following episiotomies and lacerations are critical components of postpartum care, with evidence supporting a combination of pharmacological and non-pharmacological strategies. Non-pharmacological approaches, such as cryotherapy and transcutaneous electrical nerve stimulation (TENS), have been identified as promising conservative therapies for managing early postpartum pain, offering alternatives with limited adverse effects [11]. Emerging evidence also supports the use of specific technologies. Ultimately, optimal outcomes depend on accurate diagnosis, appropriate surgical repair techniques, and the integration of these evidence-based interventions into a comprehensive, individualized care plan. A recent meta-analysis by Kurnaz et al. [18] highlights that interventions performed within the first 24 h after episiotomy did not reduce pain. However, the effects of the interventions were observed on the second day, with cold application identified as the most effective method. Additionally, interventions did not affect healing during the first three days, but a more pronounced improvement was noted in the intervention group by the fifth day. Healing began around the 7th–10th days, even without intervention. The REEDA score (redness, edema, ecchymosis, discharge, and approximation) decreased most significantly in the patients who received perineal education (diet, Kegel exercise, infection symptoms, and perineal hygiene).

Infrared therapy has been shown in phase II studies to significantly improve episiotomy wound healing and reduce pain compared to standard care alone. Constant et al. [19] compared photobiomodulation and cryotherapy in the immediate postpartum period among women with grade I and II lacerations and/or episiotomy, observing superiority of PBM in pain reduction and improved healing after 24 h. These results suggest that laser therapy can have similar or even superior results to other non-pharmacological therapies, and that dosimetry and timing of application are key determining factors in obtaining the best results. Nonetheless, data in obstetrics remain limited. The heterogeneity of protocols (including dose parameters, timing, and treatment frequency) and the lack of controlled studies comparing laser therapy with other conventional techniques have been identified as possible explanations for these inconclusive results, highlighting the need for standardization in future studies to generate higher-quality evidence and support the development of informed clinical guidelines. This study is among the first to evaluate PBM in a real-world clinical setting based on an already implemented care protocol, providing statistical evidence of its therapeutic benefit in an obstetric population with a substantial sample size of 183 women. Furthermore, statistical analysis revealed positive effects on pain and healing over three days for the group of PBM-treated patients.

### 4.1. Main Findings

This study investigated the effects of PBM on pain and perineal healing in 183 postpartum women with second- or third-degree lacerations and/or episiotomy, treated at a university hospital where PBM was already part of the standard care protocol. The results confirmed that laser application is associated with a significant reduction in pain and showed a trend toward improved healing, aligning with findings from previous studies. These outcomes are consistent with the current literature on the analgesic effects of PBM, as evidenced by a significant reduction in NPS scores both across evaluation days and immediately before and after laser application. Furthermore, pre-intervention NPS scores improved progressively across the three days, indicating a sustained benefit.

Unlike women who have not undergone any laser sessions, the results demonstrated that in the subgroup undergoing PBM, pain scores decreased throughout the hospitalization period, even though the initial NPS values of these participants were higher than those of the women who chose not to undergo the laser. Although no statistical difference was observed between women who received one laser session and those who received only conventional treatment in the propensity score matching (PSM) model, a significant difference was found between the group receiving two laser sessions and the conventional treatment group across both statistical analyses, corroborating the descriptive evolution of participants’ outcomes.

### 4.2. Interpretation

These results support the proposed mechanisms of PBM, which promote anti-inflammatory mediators and endorphin release for rapid analgesia, even in women with severe baseline pain. Furthermore, PBM represents a promising option for daily therapy in populations with chronic pain. Studies such as da Silva et al. [20] reinforce that PBM acts to modulate inflammatory processes and improve microcirculation, facilitating not only tissue repair but also providing relief from postoperative pain. Additionally, work by Nonarath et al. [21] and Rosa et al. [22] shows that PBM can reduce oxidative stress and modulate cytokine expression, which may explain the analgesic response observed in this cohort. The literature has well-documented the effects of PBM in various clinical contexts. The therapeutic effects of 808 nm PBM on pain and tissue repair are mediated by complex biomodulatory pathways. At this infrared wavelength, photons penetrate deeply into the tissue and are primarily absorbed by cytochrome c oxidase in the mitochondrial respiratory chain. This interaction enhances ATP synthesis and leads to the photodissociation of nitric oxide (NO), which promotes local vasodilation and improves oxygen delivery to the damaged tissue. For analgesia, 808 nm PBM modulates the inflammatory response by downregulating pro-inflammatory cytokines (e.g., TNF-α and IL-6) and increasing anti-inflammatory mediators, like IL-10, effectively reducing perineal edema and pressure-induced nociception. Regarding tissue regeneration, the therapy stimulates fibroblast proliferation and activates the TGF-β signaling pathway, which is essential for collagen deposition and extracellular matrix remodeling. These combined mechanisms explain the significant reduction in NPS scores and the healing rates observed, particularly in patients receiving multiple sessions. Previous studies, such as those by Chougala & Mahishale [23] and Constant et al. [19], corroborate the findings of this study, reinforcing the results of PBM for pain and perineal healing in the obstetric context.

It is important to highlight that women who chose laser therapy initially had significantly higher baseline pain (NPS scores) than those in the conventional treatment group. However, PBM application is associated with a significant reduction in pain in subsequent days, with significant pre- versus post-session differences.

Moreover, women receiving two laser sessions showed a more consistent and statistically significant pain reduction. Analysis of the three subgroups revealed that the most significant pain reduction (NPS) occurred in those receiving PBM on both days. Women treated only on day one exhibited an initial sharp reduction in pain, which diminished by day three. Those treated only on day two displayed an initial increase in pain, followed by a significant decrease after laser application. Those who received only one session did not differ significantly from controls, suggesting either a cumulative therapeutic effect associated with two PBM sessions or, as evidenced in the systematic review by Kurnaz et al. [18], an influence of the timing of laser introduction on the clinical response to pain. Regarding the results of our study, unfortunately, due to the variable sample sizes between groups and across assessment days, it was not possible to determine whether there was a difference in the evolution of participants who received laser treatment only on the first day compared to those treated only on the second day.

However, the within-group differences in the mean NPS pain scores among patients who underwent laser therapy were both statistically significant and clinically relevant. For the subgroup of patients who received a single laser session, the difference in NPS pain scores over the three-day assessment period clearly exceeded the minimal clinically important difference (MCID~2 points), although this finding was obtained from a small sample. The subgroup that received two laser sessions also demonstrated a clinically significant, albeit borderline, improvement (~1.35 points). Furthermore, at the end of follow-up, patients who received two sessions presented better outcomes than those who received no laser sessions. The main reported effect was a mean difference of 1.6761 points in NPS scores (two sessions vs. zero sessions), with a p-value of 0.000191. In terms of clinical relevance, this mean effect of 1.67 points is likely clinically meaningful and closely approaches the more conservative Minimal Clinically Important Difference (MCID) threshold (~2 points). Moreover, since the 95% confidence interval includes values above 2, it is plausible that the true (population) effect may be clinically strong in certain scenarios. In contrast, for the subgroup that received no laser sessions, the 0.75-point difference over the three days was clearly not clinically significant. These data reinforce and corroborate the statistical difference found, suggesting that the laser, in this population, is related to a greater improvement in pain than conventional treatment.

In this study, the association between lower Apgar scores and the choice of laser therapy suggests that the Apgar score may serve as a proxy for factors contributing to greater perineal pain, such as prolonged labor, instrumental delivery, or newborn size. Women with more extensive lacerations also opted more frequently for PBM, reinforcing the link between injury severity and pain levels [18].

Unfortunately, we did not carry out an analysis about other covariates such as age, type and number of previous labors, delivery type, parity, Apgar score, analgesic medications used, or laser use on the first or second day. Future post hoc analyses of these factors may clarify their influence on treatment response and provide new directions for research. We chose not to include all these adjustments in the models presented in this manuscript in order to parsimoniously preserve caution in the presentation of the results. First, increasing the number of covariates in the statistical model elevates the risk of Type I errors (false positives) due to multiple testing, particularly when these analyses were not pre-specified. Second, given the observational nature of our study and the fact that our sample size was not predetermined but rather reflected the real-world characteristics of the service and patient flow during the study period, we must exercise caution when expanding the number of covariates in our models. With a relatively small sample size, the inclusion of numerous covariates would lead to model overfitting, resulting in unstable estimates and inflated standard errors. This could produce spurious associations that are not reproducible. In observational studies with limited samples, each additional covariate reduces the degrees of freedom and increases the risk of Type I errors, particularly when these analyses were not pre-specified in a protocol. Our sample size was determined by the natural patient flow (convenience sample), meaning it provides adequate power only for the primary analyses and the limited set of covariates originally planned. Expanding the model post hoc to include multiple new covariates would violate key statistical assumptions and could generate misleading conclusions. Therefore, to maintain the statistical rigor and interpretability of our study, we have opted to retain the focus on the covariates defined in our original protocol.

For healing, REEDA scores improved most significantly in women receiving two laser sessions within the first two days. Although the PSM analysis did not reach the conventional statistical significance threshold (*p* < 0.05), a positive trend was observed at a 10% alpha level. Naturally, we acknowledge that p-values above the conventional threshold do not constitute conclusive evidence and should be interpreted with caution as suggestive findings, possibly limited by statistical power, heterogeneity of exposure, and the short observation period and sample. Moreover, the follow-up period limited to hospitalization, up to 72 h, does not allow evaluation of tissue remodeling or complete healing of second- and third-degree perineal lacerations, which in fact occur over several weeks. This assessment was primarily to analyze the early aspects of the healing process, such as early inflammatory signs, edema, ecchymosis, hyperemia, wound edge approximation, and pain evolution, which are clinically relevant parameters in the immediate postpartum period and can be captured by instruments such as the REEDA scale and the NPS. In addition, the early treatment assessment was precisely to demonstrate that there was no worsening of inflammatory signs in patients treated with PBM, as these signs may worsen in the first days in the absence of specific wound care.

We acknowledge that this short-term assessment of tissue healing should be interpreted only as an initial signal of the inflammatory phase and the early repair process rather than as an endpoint of definitive healing. A clinically meaningful time horizon for evaluating perineal healing would be a long-term follow-up until complete wound healing, since the first 24–72 h to approximately 6 weeks postpartum aligns with routine postpartum review and the expected timeline of tissue repair. Clinically relevant complications also include wound infection, suture dehiscence, persistent pain with functional impact, dyspareunia, urinary symptoms, and anorectal symptoms. Therefore, in addition to clinical instruments, such as the REEDA scale, future studies should include patient-reported outcomes, quality-of-life and treatment satisfaction questionnaires, and evaluation of return to sexual activity and urinary/anorectal symptoms. Despite these limitations, these preliminary results show a trend toward lower REEDA scores (indicating better outcomes) in the laser group, as suggested by the negative coefficient—an effect that could be confirmed in larger studies.

The last relevant aspect that impacted our results was the migration of participants between treatment subgroups throughout hospitalization, which increased data heterogeneity and analytical complexity. Despite this challenge, migration provided a rare opportunity in observational studies to compare outcomes between users and non-users of the laser therapy. This migration highlights inherent methodological challenges in pragmatic clinical studies within real-world settings, where treatment choices are guided by clinical need rather than research protocols. Although such studies require complex analysis, they generate highly applicable results by evaluating interventions under actual clinical conditions. This approach assesses not just effectiveness but also feasibility, acceptability, and impact on healthcare workflows.

Future research on the use of PBM in the postpartum period should focus on standardizing application parameters, conducting larger observational and randomized clinical trials, evaluating long-term outcomes, elucidating underlying cellular mechanisms, comparing PBM with other therapies, and analyzing cost-effectiveness. These initiatives are essential to consolidate the efficacy, safety, and applicability of PBM for pain management and perineal healing and to further support these promising results.

### 4.3. Strengths and Limitations of the Study

This study assessed PBM for pain relief and perineal healing postpartum, demonstrating that PBM is associated with a significant reduction in pain compared to conventional treatment. With a substantial sample of women, it observed both immediate effects and continuous improvement trends. While further studies on healing are needed, the results support the clinical potential of PBM and underscore the value of observational research in real-world hospital settings. By monitoring implemented protocols without artificial intervention, this study provides findings that can be directly and rapidly translated into clinical practice, enabling continuous improvements in care.

While randomized controlled trials (RCTs) operating under strictly controlled ideal conditions remain the gold standard for efficacy analysis, this ‘real-world’ observational design offers a good external validity by demonstrating the feasibility of PBM within a routine hospital workflow. This approach respects patient autonomy and pragmatic clinical practice, thereby aligning the results with clinical effectiveness. Although RCTs excel at isolating the specific effects of a therapy, our observational study contributes insights often overlooked by controlled trials, as it evaluates the intervention precisely as it occurs in a university hospital setting, facilitating a direct and rapid translation of findings into clinical practice. By allowing patients to transition between groups (migration between laser and conventional treatment), the study captures the acceptability and perceived need for the therapy among women—nuances that the forced randomization of an RCT might mask. Furthermore, whereas RCTs often employ highly restrictive inclusion criteria, this study demonstrates that PBM is effective in a diverse population with varying degrees of laceration extent and baseline pain levels. It also confirms that PBM implementation caused no adverse events and was successfully integrated with conventional therapies (analgesics and anti-inflammatories) without requiring supplemental analgesia. Moreover, to reinforce the robustness of these conclusions within a real-world setting, propensity score matching (PSM) was utilized to balance the main baseline covariates between groups, thereby minimizing the selection bias inherent in observational designs. By demonstrating that pain reduction was statistically significant even after propensity score matching, our results suggest a robust association that approaches the internal validity of an RCT while maintaining the external validity of an already implemented clinical protocol.

Of course, this study has its limitations. The NPS is a subjective measure dependent on individual perception and may be influenced by emotional, cultural, and clinical context factors, in addition to not capturing qualitative aspects of pain, such as functional or emotional impact. The REEDA scale also has a subjective component, as the assessment of items such as redness and edema may vary between examiners, potentially leading to interobserver variability. Moreover, a short REEDA scale follow-up period may have obscured more significant healing differences emerging over weeks. Furthermore, REEDA primarily evaluates superficial aspects of healing and may not fully reflect deeper tissue changes. Despite these limitations, when used in a standardized and combined manner, both scales constitute complementary and useful instruments for a comprehensive evaluation of postoperative recovery.

Other limitations involve the inability to control for natural pain resolution, potential placebo effects, and the prolonged actions of the analgesic block routinely administered during the surgical labor period. We also did not analyze the influence of other covariates—such as the type and number of previous labors, differences noted between subgroups, analgesic medications used, baseline laceration extent, or Apgar differences, as well as the timing of laser use (i.e., whether women chose treatment on the first or second day), natural recovery, placebo/context effects, and analgesic/anesthetic carryover—on outcomes. Future exploratory post hoc analyses of these factors may clarify their effects on treatment response and provide new insights for further research.

## 5. Conclusions

The results of this study demonstrate that the application of PBM to the postpartum perineum was associated with reduced pain, with a statistically significant decrease in numerical pain scale (NPS) scores over three days—even among women who initially reported higher pain levels, particularly those who received two PBM sessions. Additionally, improvement in REEDA healing scale scores was observed, especially among women who received PBM within the first 24 h after labor. Despite these promising findings, further studies are needed to confirm these effects.

## Figures and Tables

**Table 1:** Complete description of laser parameters.

Parameters	PMB
Font type	laser
Central wavelength	808 nm
Mode of operation	continuous
Average radiant power	100 mW
Radiant energy per point	4 J/cm^2^
Number of irradiated points	5, equidistant 1 cm
Anatomical location of application points	perineum
Number of sessions	2 to 3 (depending on length of hospital stay)
Session frequency	daily
Exposure time (per point)	1.2 s
Emitter output beam area	3 mm^2^
Beam area on target	3 mm^2^
Total irradiated area	15 mm^2^
Irradiance at the exit	3333 mW/cm^2^
Application technique	contact

**Table 2:** Baseline characteristics of the study sample (*n* = 183), stratified by treatment choice on the first evaluation day. For categorical variables, values are presented as the number of participants (*n*), and for continuous variables, values are presented as mean ± SD unless otherwise indicated.

Categorical Variables *	Opting for Laser	*n*	%	*p*	
Episiotomy	No	176	96.2	0.73	
	Yes	7	3.8		
Lesion location	Post.	155	84.6	0.64	
	Ant.	12	6.6		
	P. A	16	8.8		
Marital status	Single	47	25.8	0.70	
	Married	98	53.6		
	Cohabiting	38	20.7		
Education level	Primary	34	18.8	0.41	
	Secondary	134	74.0		
	Tertiary	13	7.2		
Continuous Variables **	**Opting for Laser**	***n***	**Mean**	**Standard Deviation (SD)**	***p*** **-Value**
Age (years old)	No	34	27.80	5.85	0.23
	Yes	149	25.28	5.08	
Gestational age (weeks)	No	34	38.29	5.09	0.43
	Yes	149	38.99	2.72	
Newborn weight (g)	No	34	3328.1	341	0.66
	Yes	149	3333.3	386.5	
Number of pregnancies	No	34	1.74	0.79	0.07
	Yes	149	1.5	0.86	
Apgar score at 1 min	No	34	8.77	0.65	0.01
	Yes	149	8.27	1.39	
Apgar score at 5 min	No	34	9.91	0.37	0.01
	Yes	149	9.56	0.88	
Laceration extent (cm)	No	34	2.83	0.92	0.01
	Yes	149	3.41	1.60	
Number of lesions	No	34	1.09	0.28	0.06
	Yes	149	1.20	0.51	

* Chi-square test analysis between subgroups, *p* < 0.05. ** Student’s *t*-test of statistical analysis between subgroups, *p* < 0.05.

**Table 3:** Comparison of pre-intervention pain scores (NPS) between day 1 and day 2 in the laser and no-laser participant choice on the first day. The results report within-group, between-day differences in baseline NPS (day 1 vs. day 2). Values are presented as mean ± SD, with 95% confidence.

	NPS Baseline Day 1 (*n*)		NPS Baseline Day 2 (*n*)		
Laser	Mean	S.D.	Mean	S.D.	*p*
No (*n* = 34)	1.71 (34)	2.57	1.94 (34)	2.39	0.51
IC	0.84–2.57		1.13–2.75		
Yes (*n* = 146)	4.07 (146)	2.46	3.46 * (146)	2.30	0.006 *
IC	3.67–4.47		3.09–3.83		

Repeated-measures ANOVA; *: significantly different from the pain scale on day 1.

**Table 4:** Intra-subgroups comparison of NPS scores according to the number of laser sessions. NPS scores across subgroups receiving 2, 1, or 0 laser sessions.

	Pre-NPS Day 1		Pre-NPS Day 2		Pre-NPS Day 3		
	Mean	S.D.	Mean	S.D.	Mean	S.D.	*p*
Subgroup 0 session (*n* = 12)	2.58	2.60	1.67	2.34	1.83	2.08	0.291
IC 95%	1.10–4.05		0.33–2.99		0.66–3.01		
Subgroup 1 session (*n* = 17)	3	3.29	1.74	2.30	0.94	1.78	0.016 *
IC 95%	1.43–2.57		0.67–2.86		0.09–179		
Subgroup 2 session (*n* = 73)	4.34	2.57	4.13	2.17	2.99	2.52	<0.0001 *
IC 95%	3.76–4.93		3.63–4.63		2.40–3.56		

Repeated-measures ANOVA; *: significantly different, *p* < 0.05.

**Table 5:** Comparison of pain scores (NPS) between participants with two laser sessions and those without laser after the PSM test.

NPS				
	Estimate	Std. Error	t Value	Pr (>|t|)
(Intercept)	1.2917	0.4008	3.223	0.001565
Treatment 1	1.6761	0.4379	3.828	0.000191

Residual standard error: 1.963 on 146 degrees of freedom. Multiple R-squared: 0.09121. Adjusted R-squared: 0.08499. F-statistic: 14.65 on 1 and 146 DF, *p*-value: 0.0001911.

**Table 6:** NPS scores in the subgroup that underwent laser therapy, comparing pre- and post-intervention within each treatment day. Values are presented as mean ± SD, with *p*-values and 95% confidence intervals obtained from ANOVA. The results reflect within-day changes in NPS before and after laser application for each daily session.

	Laser Day 1 (149)		Laser Day 2 (134)		Laser Day 3 (62)	
Pain Scale	Mean	Standard Deviation	Mean	Standard Deviation	Mean	Standard Deviation
NPS before	3.99	2.49	3.89	2.16	3.55	2.32
IC	3.60–4.39		3.53–4.26		2.97–4.13	
NPS after	3.06 *	2.26	2.90 *	1.99	2.56 *	2.10
IC	2.70–3.42		2.57–3.24		2.04–3.09	

Repeated-measures ANOVA; *: significantly different from pre-treatment value on the same day, *p* < 0.001.

**Table 7:** Comparison of pre-intervention REEDA scores between day 1 and day 2 in the laser and no-laser groups. The results report within-group and between-day differences in baseline REEDA (day 1 vs. day 2). Values are presented as mean ± SD, with 95% confidence intervals and *p*-values derived from repeated-measures ANOVA.

	REEDA Baseline Day 1 (*n*)		REEDA Baseline Day 2 (*n*)	
Laser	Mean	S.D.	Mean	S.D.
No (*n* = 34)	1.26 (34)	0.79	1.35 (34)	0.81
IC	0.99–1.53		1.08–1.62	
Yes (*n* = 146)	1.82 (146)	0.97	1.42 * (146)	0.89
IC	1.66–197		1.28–1.57	

Repeated-measures ANOVA; *: significantly different from REEDA on day 1, *p* < 0.0001.

## Data Availability

The original contributions presented in this study are included in the article/Supplementary Material. Further inquiries can be directed to the corresponding author(s).

## Supplementary Materials

The following supporting information can be downloaded at: https://www.mdpi.com/article/10.3390/medsci14010125/s1, Figure S1: Mean NPS values before the intervention over the three days offollow-up among subgroups receiving 0, 1, or 2 laser sessions during the threedays of hospitalization; Figure S2: Mean REEDA values before the intervention over the three days of follow-up amongsubgroups receiving 0, 1, or 2 laser sessions during the three days of hospitalization; Table S1: Comparison of pain scores (NPS) between participants with one laser sessions and those without laser after PSM test; Table S2: Comparison of pain scores (NPS) between participants with two laser sessions and those with one laser after PSM test; Table S3: Inter-subgroup comparison of REEDA scores according to the number of laser sessions. REEDA scores across subgroups receiving 2, 1, or 0 laser sessions; Table S4: Comparison of REEDA Scores between participants with two laser sessions and those without laser after PSM test; Table S5: Comparison of REEDA scores between participants with one laser sessions and those without laser after PSM test; Table S6: Comparison of REEDA scores between participants with one laser sessions and those with two laser after PSM test; and Supplementary File S1: Statistical Analysis Plan.

## Abbreviations

The following abbreviations are used in this manuscript:

CAPES	Coordenação de Aperfeiçoamento de Pessoal de Nível Superior
cm	Centimeter
HUMAI-UEPG	Hospital Universitário Materno-Infantil Universidade Estadual Ponta-Grossa
J	Joule
mW	miliWatt
NPS	Numerical Pain Scale
PBM	Photobiomodulation
PI	Principal Investigator
PROEX	Programa de Excelência Acadêmica
PSM	Propensity Score Matching
W	Watt
