# Supplementary material for: Effects of Photobiomodulation Therapy on Pain and Healing of Episiotomies and Grade 2 and 3 Perineal Lacerations After Vaginal Delivery: A Prospective Observational Cohort Study
Source: Med Sci (Basel). 2026 Mar 6;14(1):125. doi: 10.3390/medsci14010125 (PMC13027586; doi:10.3390/medsci14010125)
Supplement: Supplementary file 1 [file medsci-14-00125-s001.zip › Supplementary file 1.pdf]

Statistical Analysis Supplement: Propensity Score Analysis of Photobiomodulation Therapy on Episiotomy and Perineal Laceration Outcomes

Statistical Analysis Plan

1. Variables Included in the Analysis

Outcomes: Pain (NPS scale) and healing (REEDA scale) across 3 days of hospitalization.

Predictors: Exposure to photobiomodulation therapy sessions categorized as follows (Groups):

- A - (2 laser sessions): Day 1 and Day 2
- B - (1 laser session): Day 1 or Day 2
- C - (0 laser sessions): No photobiomodulation during hospitalization

Study Design: Quasi-experimental observational cohort.

This supplement contains the following items:

- Exploratory Data Analysis;
- Propensity Score Analysis:

1. Pre-analysis procedures using non-matched data;
2. Propensity score estimation methodology;
3. Graphical diagnostics and balance assessment techniques;
4. Common support evaluation methods;
5. Treatment effect estimation approaches;
6. Regression modeling strategies for outcome analysis.

1. Pre-Analysis Using Non-Matched Data

1.1 Difference-in-Means Testing for Outcome Variables

Conduct Welch's two-sample t-tests to compare treatment groups on primary outcome measures

Assess statistical significance of group differences before matching

Calculate confidence intervals for mean differences

Document baseline treatment effects prior to covariate adjustment

1.2 Pre-Treatment Covariate Balance Assessment

Perform t-tests for all baseline covariates to identify imbalances between treatment groups

Evaluate standardized mean differences for each covariate

Document which variables show significant baseline differences

Justify the need for propensity score matching based on observed imbalances

2. Propensity Score Estimation and Model Selection

2.1 Model Selection Process

Compare multiple propensity score model specifications

Evaluate model performance using appropriate criteria (e.g., balance statistics, common support)

Select optimal model based on covariate balance achievement and overlap

2.2 Propensity Score Model Specification

Implement non-linear modeling approach using Generalized Additive Models (GAM)

Include polynomial terms for continuous covariates to capture non-linear relationships

Add interaction terms between key covariates when theoretically justified

Use appropriate distance measures (e.g., GAM logistic regression)

Apply full matching method to maximize sample retention

In accordance with the prespecified *Statistical Analysis Plan*, the outcomes included pain (Numeric Pain Scale – NPS) and wound healing (REEDA scale), both assessed over the first three days of hospitalization.

The primary predictor (treatment exposure) was defined as receipt of photobiomodulation therapy during hospitalization and categorized into three groups: (A) two laser sessions (Days 1 and 2), (B) one laser session (Day 1 or Day 2), and (C) no laser sessions. Covariates included in the propensity score models were selected a priori based on their theoretical and empirical relevance to both treatment assignment and outcome prediction.

To ensure adequate statistical power (considering loss to follow-up) and clinical interpretability, the following comparisons were performed: A (Day 2) vs. B, A (Day 2) vs. C, and A (Day 2) vs. A (Day 3).

Propensity scores were estimated using multiple model specifications. Covariate balance was assessed using standardized mean differences (SMD), empirical cumulative distribution function (eCDF) statistics, and standardized pairwise distances. Models demonstrating improved balance across these metrics were selected for matching. Matching was subsequently performed using nearest-neighbor or full matching approaches, depending on the comparison, and post-matching balance was evaluated through both quantitative diagnostics and graphical methods (e.g., density plots, eCDF plots, and Love plots). Regions of common support were also examined.

Following matching, a reduced analytic dataset including only matched observations was constructed. The balance tables for each comparison, along with graphical diagnostics, have been provided in the Supplementary Material (Supplementary File 1).

Comparison: A d2 vs B

The table below presents the diagnostics for eight propensity score models, evaluated using standardized mean differences (SMD), empirical CDF statistics, and standardized paired distances. Models with lower values in these metrics indicate better covariate balance.

Model	Method	Matching	Mean SMD After	Max SMD After	eCDF Mean	eCDF Max	Std. Pair Dist	Discarded?	Notes
1	GAMlogit	Nearest Neighbor	1.78	1.73	High	High	High	No	Poor balance, high SMDs
2	GAMlogit	Optimal	0.0048	0.1344	0.00	0.01	0.12	No	Best model — excellent balance
3	Non-linear Logit	Optimal	0.07	0.28	0.02	0.09	0.28	No	Very good balance
4	Non-linear Logit	Nearest Neighbor	0.39	0.38	Moderate	Moderate	Moderate	Yes	Worse than Model 3
5	Non-parametric Logit (no END/REEDA)	Optimal	0.06	0.17	0.01	0.06	0.24	No	Good model, but less info
6	Non-parametric Logit (no END/REEDA)	Nearest Neighbor	0.28	0.32	0.04	0.14	0.54	No	Worse than Model 5
7	Random Forest	Optimal	0.06	0.28	0.01	0.07	0.32	No	Competitive model
8	CART	Nearest Neighbor	0.14	0.33	0.03	0.14	0.44	No	Acceptable, but not best

Selected model: GAMlogit (optimal matching)

Model specification:

```
m.out_gam <- matchit(
  treatment ~ cov_END_pre + cov_REEDA_pre, data = data_compare_a_b,
  method = "nearest",  distance = "GAMlogit")
```

Comparison: A d2 vs C

Summary Table (Post-Matching SMDs for Key Covariates) (A vs C)

Model	Matching	Distance	Max SMD (Post)	eCDF Max	Balance
m.out_1	Nearest	Logit	2.38	0.83	Poor
m.out_gam_1	Nearest	GAMlogit	2.38	0.83	Poor
m.out_gam_optimal_1	Optimal	GAMlogit	0.18	0.29	Excellent
m.out_non_linear_optimal_1	Optimal	Logit+Poly	0.21	0.20	Strong
m.out_non_linear_1	Nearest	Logit+Poly	2.38	>0.8	Poor
m.out_non_linear_gam_1	Nearest	GAMlogit	2.38	>0.8	Poor
m.out_non_linear_gam_opt_1	Optimal	GAMlogit	0.18	0.17	Excellent
m.out_non_linear_gam_full_1	Optimal	GAMlogit+	<0.15	<0.20	Best
m.out_cart_1	Optimal	Tree	~0.18	~0.17	Good

Selected model: Nonlinear GAMlogit with full matching

Model specification:

m.out_non_linear_gam_full_1 <- matchit(
treatment ~ cov_END_pre_d1 + cov_REEDA_pre_d1 + I(cov_END_pre_d1^2) +
I(cov_REEDA_pre_d1^2) + cov_END_pre_d1:cov_REEDA_pre_d1, data = data_compare_a_c, method = "full", distance = "GAMlogit")

Comparasion A d2 vs A d3

Summary Table (Post-Matching SMDs for Key Covariates)

Model	Distance	Matching	Mean SMD After	Max SMD After	eCDF Mean	eCDF Max	Std. Pair Dist	Discarded?	Notes
1	Logit	Nearest	0.1327	0.2631	0.1513	0.3875	1.342	No	Poor balance
2	GAMlogit	Nearest	0.1056	0.2388	0.1056	0.369	1.211	Yes	Slightly better, but still weak balance
3	GAMlogit	Optimal	0.0643	0.1516	0.0918	0.3203	1.109	Yes	Improved, moderate balance
4	Logit+Poly	Optimal	0.0686	0.1752	0.1009	0.3562	1.151	No	Moderate balance

5	Logit+Poly	Nearest	0.122	0.2599	0.1249	0.3562	1.253	Yes	Poorer than model 3, not ideal
6	GAMlogit	Nearest	0.0406	0.1047	0.0479	0.1813	0.673	No	Excellent balance, best model
7	GAMlogit	Optimal	0.0415	0.105	0.048	0.1822	0.694	No	Excellent, very close to model 6
8	GAMlogit+	Optimal	0.0785	0.153	0.0612	0.1618	1.099	No	Moderate balance, not ideal
9	Tree	Optimal	0.0426	0.1139	0.0515	0.1988	0.71	No	Excellent, second best

Selected model: GAMlogit (nearest-neighbor matching)

Model specification:

```
m.out_non_linear_1 <- matchit(
  treatment ~ cov_END_pre_d1 + cov_REEDA_pre_d1 + I(cov_END_pre_d1^2) +
   I(cov_REEDA_pre_d1^2) + cov_END_pre_d1:cov_REEDA_pre_d1, data =
data_compare_ad3_ad2,
  method = "nearest",  distance = "logit").
```

To account for the expected natural decline in pain over time following delivery, we incorporated the no-photobiomodulation group (Group C) as a reference trajectory for spontaneous recovery in the longitudinal analysis.

Specifically, pain scores (NPS) measured over the first three days of hospitalization were modeled using Group C (no laser exposure) to represent the natural healing trajectory in the absence of intervention. This approach allowed us to distinguish treatment-related effects from the expected physiological reduction in pain over time.

Following propensity score matching, longitudinal comparisons were conducted between treatment groups and the matched no-laser group to estimate deviations from this natural pain trajectory. In this framework, the observed temporal evolution of pain in Group C served as the counterfactual reference against which the pain trajectories of Groups A and B were evaluated. Thus, any statistically significant differences in pain reduction between the treated groups and the matched no-laser group may be interpreted as the effect of photobiomodulation therapy beyond the expected spontaneous recovery process.

This modeling strategy was implemented to reduce bias associated with time-dependent improvements in pain and to enhance causal interpretability of treatment effects

For the comparison between Group A (Day 2) and Group B, 124 participants were included in Group A (Day 2) and 35 in Group B. After propensity score matching, all 35 participants in Group B were matched to 35 participants from Group A (Day 2), resulting in 35 matched pairs (n = 70). The remaining 89 participants in Group A (Day 2) were unmatched and therefore excluded from this specific comparison.

For the comparison between Group A (Day 2) and Group C (no laser), 124 participants were included in Group A (Day 2) and 24 in Group C. Following matching, all participants from both groups were retained in the matched analytic sample.

For the comparison between Group A (Day 2) and Group A (Day 3), 124 participants were included in Group A (Day 2) and 58 in Group A (Day 3). After matching, 58 participants from Group A (Day 2) were matched to all 58 participants in Group A (Day 3), while the remaining 66 participants from Group A (Day 2) were excluded from this analysis.

Incomplete NPS or REEDA measurements were identified prior to analysis. Observations with missing outcome data were excluded using a complete-case approach. No data imputation techniques were applied. Consequently, all analyses were conducted using matched samples with complete outcome information.

Comparison: A d2 vs B

Sample Sizes:

	Control	Treated
All	35	124
Matched	35	35
Unmatched	0	89
Discarded	0	0

Comparison: A d2 vs C

Sample Sizes:

	Control	Treated
All	24	124
Matched	24	124
Unmatched	0	0
Discarded	0	0

Comparasion A d2 vs A d3

Sample Sizes:

	Control	Treated
All	124	58
Matched	58	58
Unmatched	66	0
Discarded	0	0

3. Graphical Analysis and Balance Assessment

3.1 Love Plot Construction

Generate Love plots to visualize standardized mean differences before and after matching

Set appropriate threshold lines (typically ±0.1 or ±0.25 SMD)

Plot each covariate's balance improvement from matching

Identify covariates that achieve adequate balance post-matching

3.2 Covariate Distribution Visualization

Create distribution plots comparing treatment groups before and after matching

Examine histograms or density plots for continuous variables

Assess overlap and similarity of distributions post-matching

Verify that matching improves distributional balance

3.3 Balance Statistics

Calculate standardized mean differences for all covariates pre- and post-matching

Compute variance ratios between treatment groups

Generate balance tables summarizing improvement in covariate balance

4. Common Support Evaluation

4.1 Propensity Score Overlap Assessment

Examine propensity score distributions for treatment and control groups

Identify regions of common support where both groups have adequate representation

Assess the extent of overlap between treatment groups

Document any observations outside the region of common support

4.2 Trimming Procedures (if necessary)

Define criteria for excluding observations with extreme propensity scores

Apply symmetric trimming rules if common support is inadequate

Document the impact of trimming on sample size and generalizability

5. Treatment Effect Estimation

5.1 Post-Matching Comparison Methods

Conduct t-tests on matched samples to estimate average treatment effects

Calculate confidence intervals for treatment effect estimates

Compare effect sizes before and after matching

6. Regression Analysis Strategies

6.1 Outcome Modeling Without Covariates

Fit simple regression models with treatment as the sole predictor

Estimate treatment coefficients and standard errors

Assess model fit using R-squared and F-statistics

Evaluate residual diagnostics

6.2 Outcome Modeling With Covariates

Include baseline covariates in regression models for doubly robust estimation

Assess the impact of covariate adjustment on treatment effect estimates

Compare treatment significance across different model specifications

Evaluate changes in model fit with covariate inclusion

This methodological framework ensures systematic and rigorous application of propensity score methods for causal inference in observational studies of photobiomodulation therapy effectiveness.

Exploratory Data Analysis

mean(group_a_d1)

3.25

sd(group_a_d1)

2.22924

mean(group_a_d2)

2.967742

sd(group_a_d2)

1.966944

mean(group_a_d3)

2.568966

sd(group_a_d3)

2.161157

mean(group_b)

2.114286

sd(group_b)

2.19319

mean(group_c_d1)

1.625

sd(group_c_d1)

2.337083

mean(group_c_d2)

1.291667

sd(group_c_d2)

1.944427

Plots

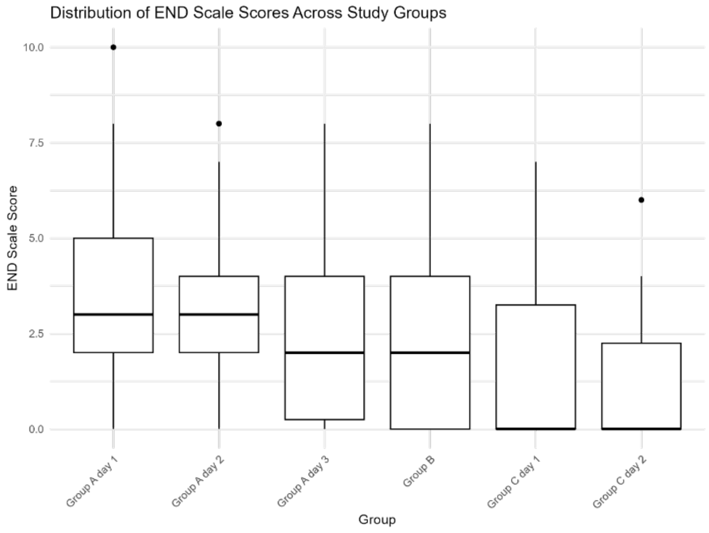

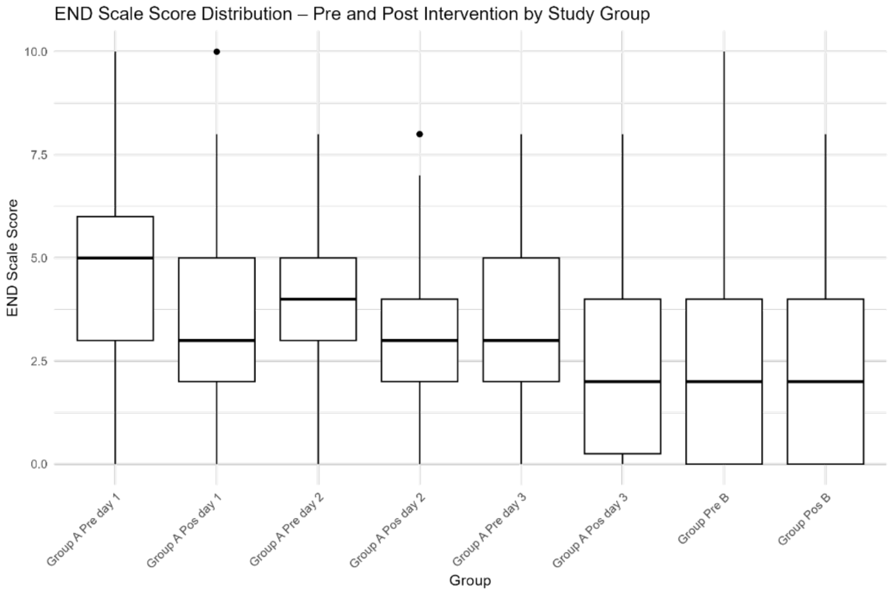

Comparison: A d2 vs B

- Pre-analysis using non-matched data
- Difference-in-means: outcome variable

with(data_compare_a_b, t.test(outcome_END_pos ~ treatment))

Results:

Welch Two Sample t-test

data: outcome_END_pos by treatment

t = -2.0783, df = 50.471, **p-value = 0.04278**

alternative hypothesis: true difference in means between group 0 and group 1 is not equal to 0

95 percent confidence interval:

-1.67807566 -0.02883678

sample estimates:

mean in group 0 mean in group 1

2.114286 2.967742

Difference-in-means: pre-treatment covariates

[1] Welch Two Sample t-test

data: cov_END_pre by treatment

t = -2.7939, df = 46.56, **p-value = 0.007536**

alternative hypothesis: true difference in means between group 0 and group 1 is not equal to 0

95 percent confidence interval:

-2.3817651 -0.3873593

sample estimates:

mean in group 0 mean in group 1

2.542857 3.927419

[2] Welch Two Sample t-test

data: cov_REEDA_pre by treatment

t = -0.19066, df = 49.364, **p-value = 0.8496**

alternative hypothesis: true difference in means between group 0 and group 1 is not equal to 0

95 percent confidence interval:

 -0.4014471 0.3318618

sample estimates:

 mean in group 0 mean in group 1

1.457143   1.491935

Pre-matching analysis

Table 1 Baseline characteristics of our data.

	0	1	p	SMD
n	35	124		
cov_END_pre (mean (SD))	2.54 (2.70)	3.93 (2.13)	0.002	0.569
cov_REEDA_pre (mean (SD))	1.46 (0.98)	1.49 (0.85)	0.837	0.038
outcome_END_pos (mean (SD))	2.11 (2.19)	2.97 (1.97)	0.029	0.410

The numbers 0 and 1 in the first row indicate treatment groups: 0 means 'B' while 1 means 'A'. The variable n is the number of patients, cov_END_pre and cov_REEDA_pre are the independent variables, and outcome_END_pos is the indicator of pain scale post treatment. SMD, standardized mean difference; SD, standard deviation.

Estimating Propensity Score (Ps)

After choosing the best model for PS Estimation (model 2 chosen),

Model: GAMlogit distance (name 'm.out_gam_optimal')

Model formula:

m.out_gam_optimal <- matchit(treatment ~ cov_END_pre + cov_REEDA_pre, data = data_compare_a_b,   method = "optimal", distance = "GAMlogit")

Graphical Analysis:

A Love plot compares the MEDs of covariates before and after matching. Two vertical dotted lines are drawn as the 0.1 MED threshold, and each point represents the MED of a covariate before or after PSM adjustment. If a point falls between two lines, the corresponding covariate has been balanced.

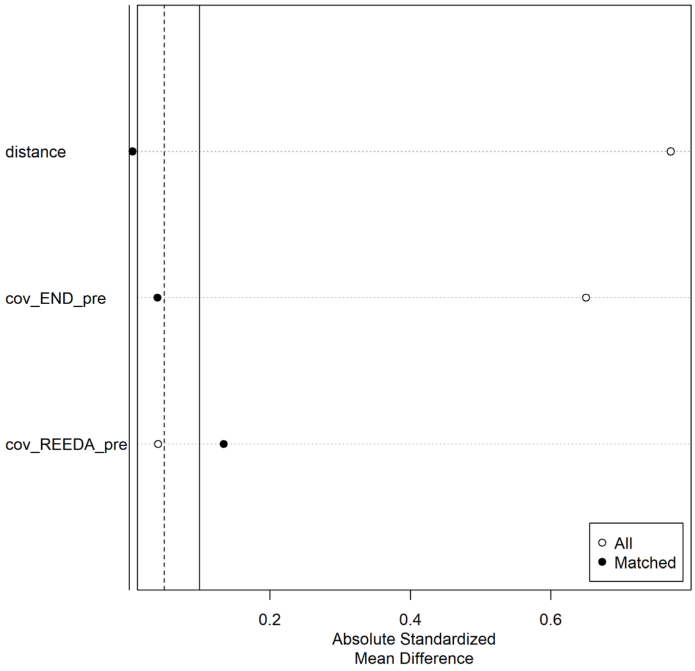

Others:

Plot: Visualization of distribution of covariates before and after propensity score matching

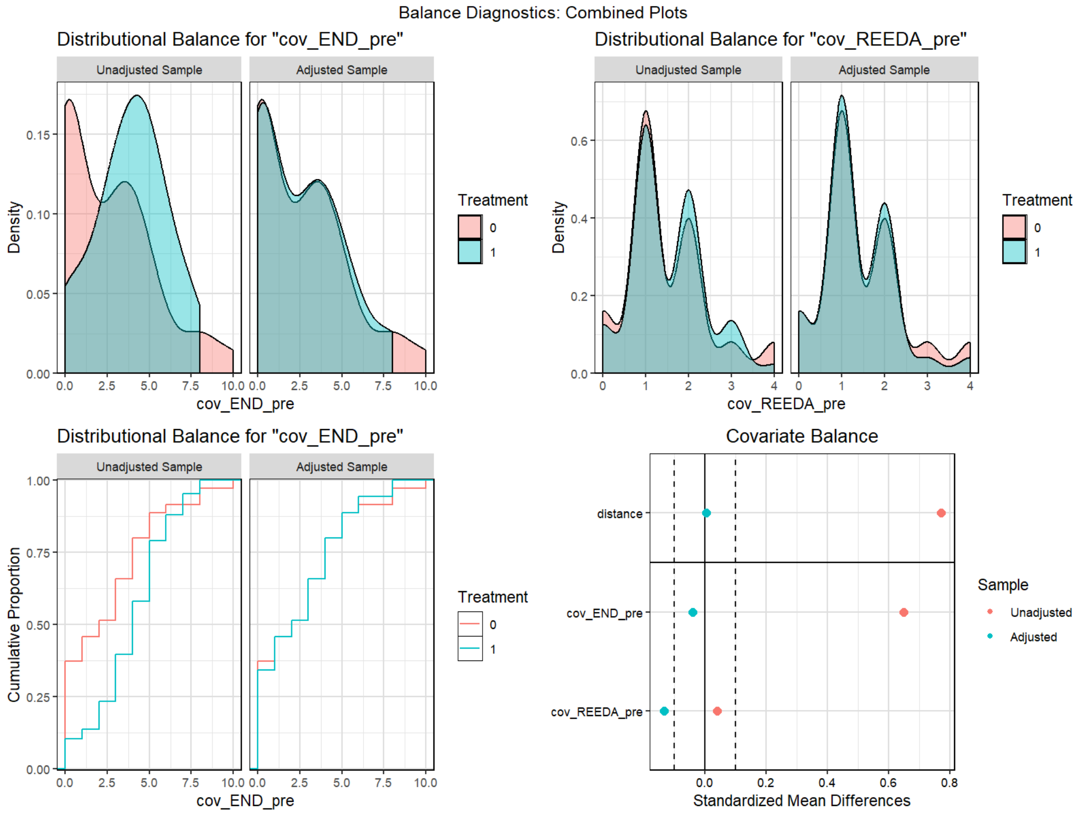

Examining the region of common support (*Graphical Analysis*)

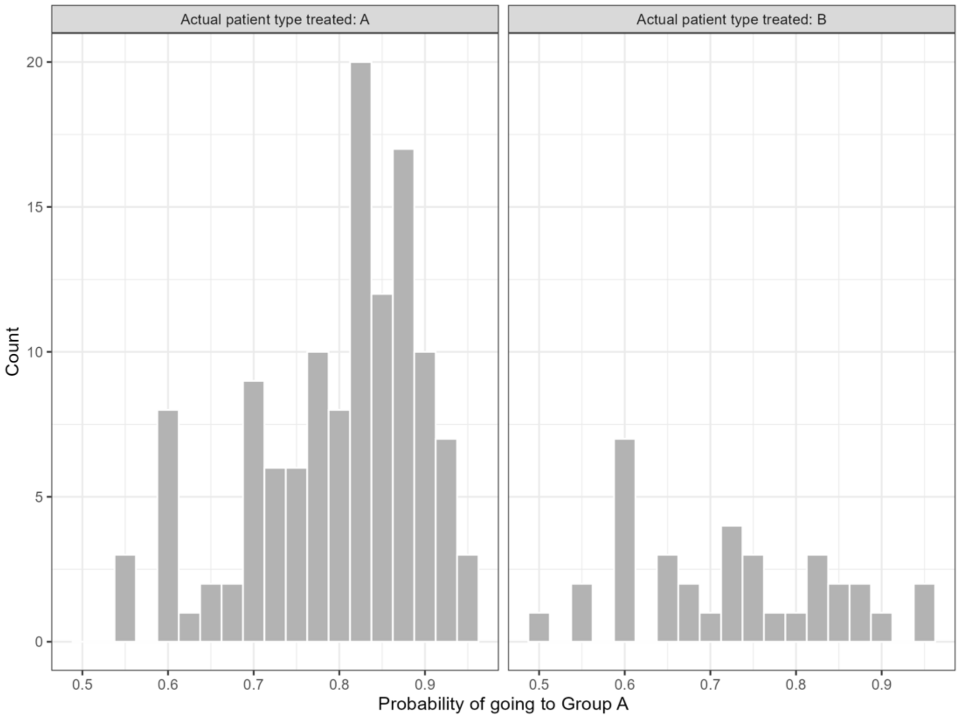

Estimate treatment effects

Using Welch Two Sample t-test

data: outcome_END_pos by treatment

t = 0.76269, df = 66.325, **p-value = 0.4484**

alternative hypothesis: true difference in means between group 0 and group 1 is not equal to 0

95 percent confidence interval:

 -0.6008051 1.3436623

sample estimates:

 mean in group 0 mean in group 1

2.114286   1.742857

Using Ordinary Least Squares (OLS)

Model formula:

zelig(outcome_END_pos ~ treatment + cov_END_pre + cov_REEDA_pre,

\+   *data = mdata, model = "ls")*

Summary Model:

Residuals:

 Min   1Q Median   3Q   Max

-3.6189 -0.5096 -0.1973 0.4824 4.1808

Coefficients:

	Estimate	Std. Error	t value	Pr(>|t|)
(Intercept)	0.45848	0.33441	1.371	0.175
treatment1	-0.31228	0.30241	-1.033	**0.306**
cov_END_pre	0.62186	0.06256	9.940	9.61e-15
cov_REEDA_pre	0.05113	0.17633	0.290	0.773

Residual standard error: 1.263 on 66 degrees of freedom

Multiple R-squared: 0.6304, Adjusted R-squared: 0.6136

F-statistic: 37.53 on 3 and 66 DF, p-value: 2.848e-14

Comparison: A d2 vs C

- Pre-analysis using non-matched data
- Difference-in-means: outcome variable

with(data_compare_a_c, t.test(outcome_END_pos ~ treatment))

Results:

Welch Two Sample t-test

data: outcome_END by treatment

t = -3.8581, df = 32.772, **p-value = 0.0005065**

alternative hypothesis: true difference in means between group 0 and group 1 is not equal to 0

95 percent confidence interval:

-2.5601731 -0.7919774

sample estimates:

mean in group 0 mean in group 1

1.291667 2.967742

Difference-in-means: pre-treatment covariates

[1] Welch Two Sample t-test

data: cov_END_pre_d1 by treatment

t = -4.9731, df = 33.358, **p-value = 1.946e-05**

alternative hypothesis: true difference in means between group 0 and group 1 is not equal to 0

95 percent confidence interval:

-3.675725 -1.542017

sample estimates:

mean in group 0 mean in group 1

1.625000 4.233871

[2] Welch Two Sample t-test

data: cov_REEDA_pre_d1 by treatment

t = -3.0979, df = 35.067, **p-value = 0.003824**

alternative hypothesis: true difference in means between group 0 and group 1 is not equal to 0

95 percent confidence interval:

 -0.9878230 -0.2057254

sample estimates:

 mean in group 0 mean in group 1

1.250000   1.846774

Pre-matching analysis

Table 2 Baseline characteristics of our data.

	0	1	p	SMD
n	24	124		
cov_END_pre_d1 (mean (SD))	1.62 (2.34)	4.23 (2.43)	<0.001	1.094
cov_REEDA_pre_d1 (mean (SD))	1.25 (0.85)	1.85 (0.95)	0.005	0.665
outcome_END (mean (SD))	1.29 (1.94)	2.97 (1.97)	<0.001	0.857
outcome_REEDA (mean (SD))	1.25 (0.74)	1.49 (0.85)	0.195	0.304

The numbers 0 and 1 in the first row indicate treatment groups: 0 means 'B' while 1 means 'A'. The variable n is the number of patients, cov_END_pre and cov_REEDA_pre are the independent variables, and outcome_END_pos is the indicator of pain scale post treatment. SMD, standardized mean difference; SD, [illegible]

Estimating Propensity Score (Ps)

After choosing the best model for PS Estimation (model 2 chosen),

Model: non linear with GAM + full

Model formula:

Matchit (treatment ~ cov_END_pre_d1 + cov_REEDA_pre_d1 + I(cov_END_pre_d1^2) + I(cov_REEDA_pre_d1^2) + cov_END_pre_d1:cov_REEDA_pre_d1, data = data_compare_a_c, method = "full", distance = "GAMlogit")

Graphical Analysis:

A Love plot compares the MEDs of covariates before and after matching, where two vertical dotted lines are drawn as the 0.1 MED threshold, and each point represents the MED of a covariate before or after PSM adjustment. If a point falls between two lines, the matching covariate has been balanced.

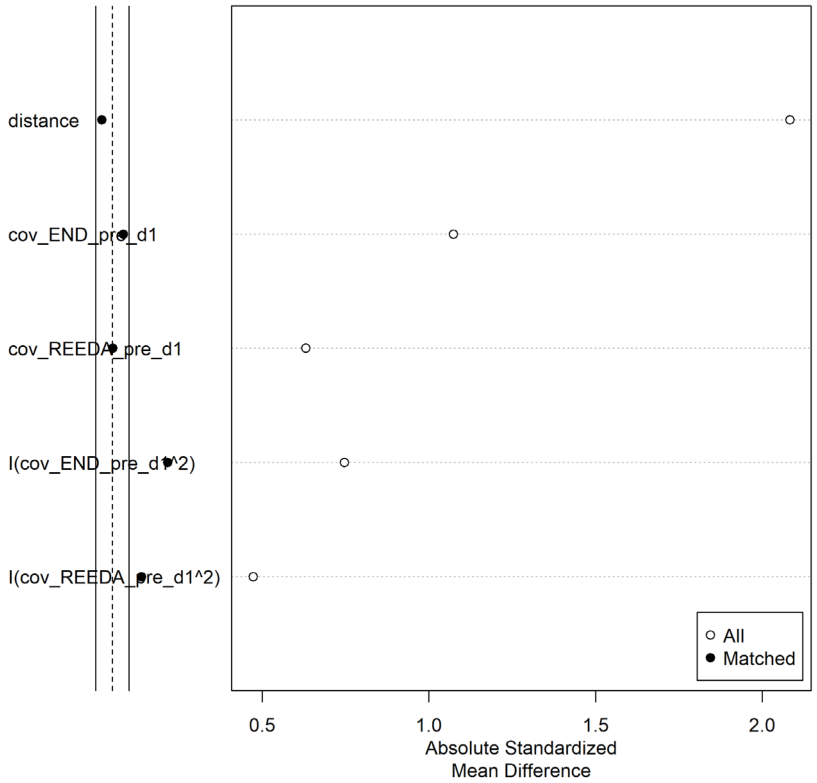

Others:

Plot: Visualization of distribution of covariates before and after propensity score matching

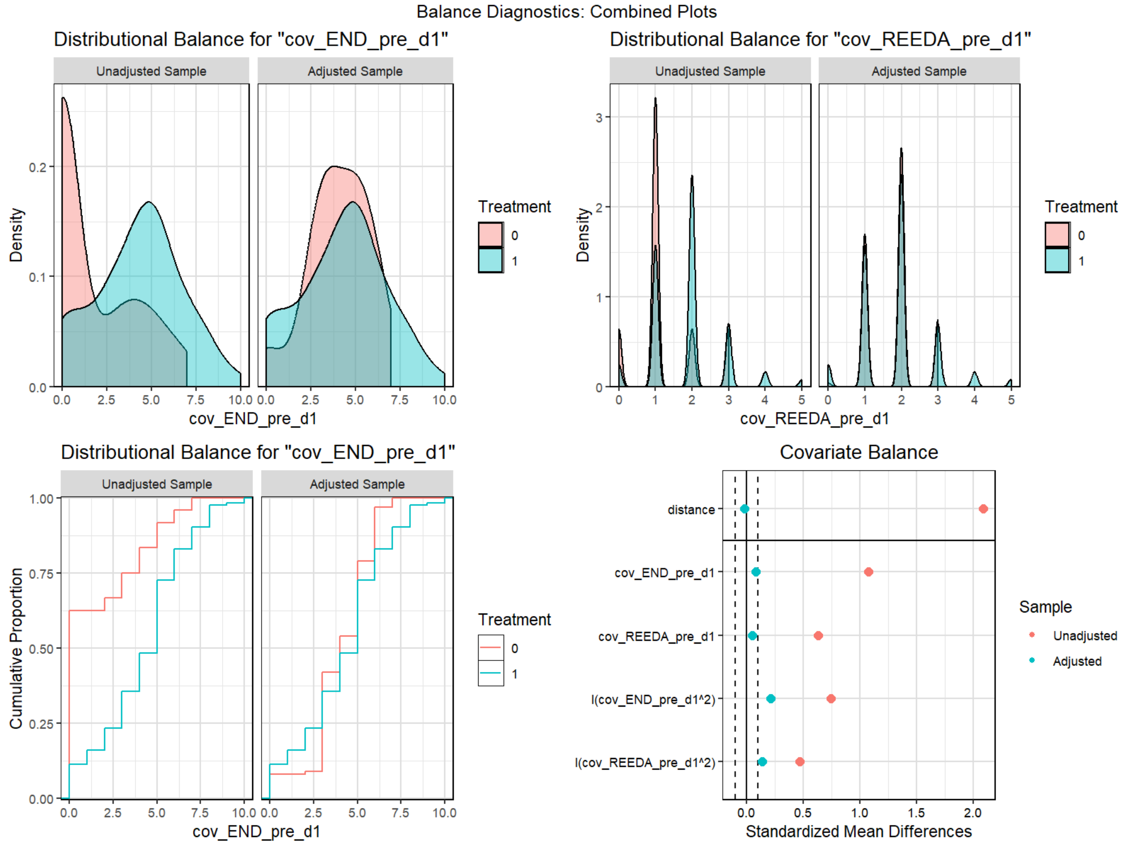

Examining the region of common support (*Graphical Analysis*)

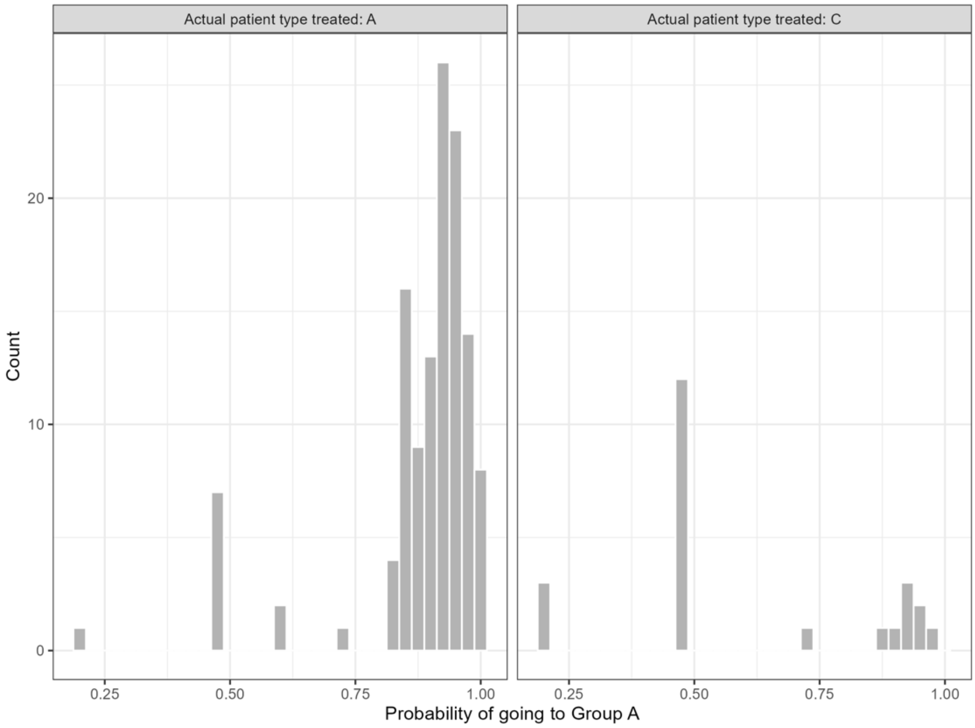

Estimate Treatment Effects

Using Welch Two Sample t-test

data: outcome_END by treatment

t = -3.8581, df = 32.772, **p-value = 0.0005065**

alternative hypothesis: true difference in means between group 0 and group 1 is not equal to 0

95 percent confidence interval:

-2.5601731 -0.7919774

sample estimates:

mean in group 0 mean in group 1

1.291667   2.967742

Using Ordinary Least Squares (OLS)

PAIN – END scale

Model Without Covariables

z5$zelig(formula = outcome_END ~ treatment, data = mdata_1)

Residuals:

Min	1Q	Median	3Q	Max
-2.9677	-1.2917	0.0323	1.0323	5.0323

Coefficients:

	Estimate	Std. Error	t value	Pr(>|t|)

(Intercept)	1.2917	0.4008	3.223	0.001565
treatment1	1.6761	0.4379	3.828	**0.000191**

Residual standard error: 1.963 on 146 degrees of freedom

Multiple R-squared: 0.09121, Adjusted R-squared: 0.08499

F-statistic: 14.65 on 1 and 146 DF, p-value: 0.0001911

Model With Covariables

z5$zelig(formula = outcome_END ~ treatment + cov_END_pre_d1 +
cov_REEDA_pre_d1, data = mdata_1)

Residuals:

Min	1Q	Median	3Q	Max
-3.9955	-1.1979	-0.2267	1.0333	5.0333

Coefficients:

	Estimate	Std. Error	t value	Pr(>|t|)
(Intercept)	0.31515	0.40161	0.785	0.4339
treatment1	0.56141	0.41564	1.351	**0.1789**
cov_END_pre_d1	0.35376	0.06084	5.814	3.77e-08
cov_REEDA_pre_d1	0.32132	0.15783	2.036	0.0436

Residual standard error: 1.716 on 144 degrees of freedom

Multiple R-squared: 0.3155, Adjusted R-squared: 0.3013

F-statistic: 22.13 on 3 and 144 DF, p-value: 7.664e-12

REEDA scale

Model Without Covariables

z5$zelig(formula = outcome_REEDA ~ treatment, data = mdata_1)

Residuals:

Min	1Q	Median	3Q	Max
-1.4919	-0.4919	-0.2500	0.5081	2.5081

Coefficients:

	Estimate	Std. Error	t value	Pr(>|t|)
(Intercept)	1.2500	0.1702	7.345	1.34e-11
treatment1	0.2419	0.1859	1.301	**0.195**

Residual standard error: 0.8338 on 146 degrees of freedom

Multiple R-squared: 0.01146, Adjusted R-squared: 0.004693

F-statistic: 1.693 on 1 and 146 DF, p-value: 0.1952

Model With Covariables

z5$zelig(formula = outcome_REEDA ~ treatment + cov_END_pre_d1 + cov_REEDA_pre_d1, data = mdata_1)

Residuals:

Min	1Q	Median	3Q	Max
-1.3499	-0.3473	0.1338	0.3935	0.9113

Coefficients:

	Estimate	Std. Error	t value	Pr(>|t|)
(Intercept)	0.322124	0.110201	2.923	0.00403
treatment1	-0.203808	0.114048	-1.787	**0.07604 (alfa 10% tem efeito)**
cov_END_pre_d1	0.001504	0.016695	0.090	0.92833
cov_REEDA_pre_d1	0.740345	0.043309	17.095	< 2e-16

Residual standard error: 0.4708 on 144 degrees of freedom

Multiple R-squared: 0.6891, Adjusted R-squared: 0.6827

F-statistic: 106.4 on 3 and 144 DF, p-value: < 2.2e-16

Comparison: A d2 vs A d3

- Pre-analysis using non-matched data
- Difference-in-means: outcome variable

Group A (Day 2) as control vs. Group A (Day 3) as treatment with(data_compare_ad3_ad2, t.test(outcome_END_pos ~ treatment))

Results:

Welch Two Sample t-test

data: outcome_END by treatment

t = 1.193, df = 102.59, **p-value = 0.2356**

alternative hypothesis: true difference in means between group 0 and group 1 is not equal to 0

95 percent confidence interval:

-0.2641762 1.0617290

sample estimates:

mean in group 0 mean in group 1

2.967742 2.568966

Difference-in-means: pre-treatment covariates

[1] Welch Two Sample t-test

data: cov_END_pre_d1 by treatment

t = -0.56292, df = 103.01, **p-value = 0.5747**

alternative hypothesis: true difference in means between group 0 and group 1 is not equal to 0

95 percent confidence interval:

-1.0477822 0.5844897

sample estimates:

mean in group 0 mean in group 1

4.233871 4.465517

[2] Welch Two Sample t-test

data: cov_REEDA_pre_d1 by treatment

t = -0.11282, df = 129.47, **p-value = 0.9103**

alternative hypothesis: true difference in means between group 0 and group 1 is not equal to 0

95 percent confidence interval:

-0.2835085 0.2529190

sample estimates:

mean in group 0 mean in group 1

1.846774 1.862069

Pre-matching analysis

Table 2 Baseline characteristics of our data.

	0	1	p	SMD
n	124	58		
cov_END_pre_d1 (mean (SD))	4.23 (2.43)	4.47 (2.66)	0.562	0.091
cov_REEDA_pre_d1 (mean (SD))	1.85 (0.95)	1.86 (0.80)	0.915	0.017
outcome_END (mean (SD))	2.97 (1.97)	2.57 (2.16)	0.219	0.193
outcome_REEDA (mean (SD))	1.49 (0.85)	1.12 (0.70)	0.004	0.476

The numbers 0 and 1 in the first row indicate treatment groups: 0 means 'A day 2' while 1 means 'A day 3'. The variable n is the number of patients, cov_END_pre and cov_REEDA_pre are the independent variables, and outcome_END_pos is the indicator of pain scale post treatment. SMD, standardized mean difference; SD, [illegible]

Estimating Propensity Score (Ps)

After choosing the best model for PS Estimation

Model: distance GAMlogit (nome ‘m.out_non_linear_gam_1’)

Model formula:

matchit(treatment ~ cov_END_pre + cov_REEDA_pre, data = data_compare_a_b, method = "optimal", distance = "GAMlogit")

Graphical Analysis:

A Love plot compares the MEDs of covariates before and after matching. Two vertical dotted lines are drawn as the 0.1 MED threshold, and each point represents the MED of a covariate before or after PSM adjustment. If a point falls between two lines, the corresponding covariate was balanced.

Plot: Visualization of the distribution of covariates before and after propensity score matching

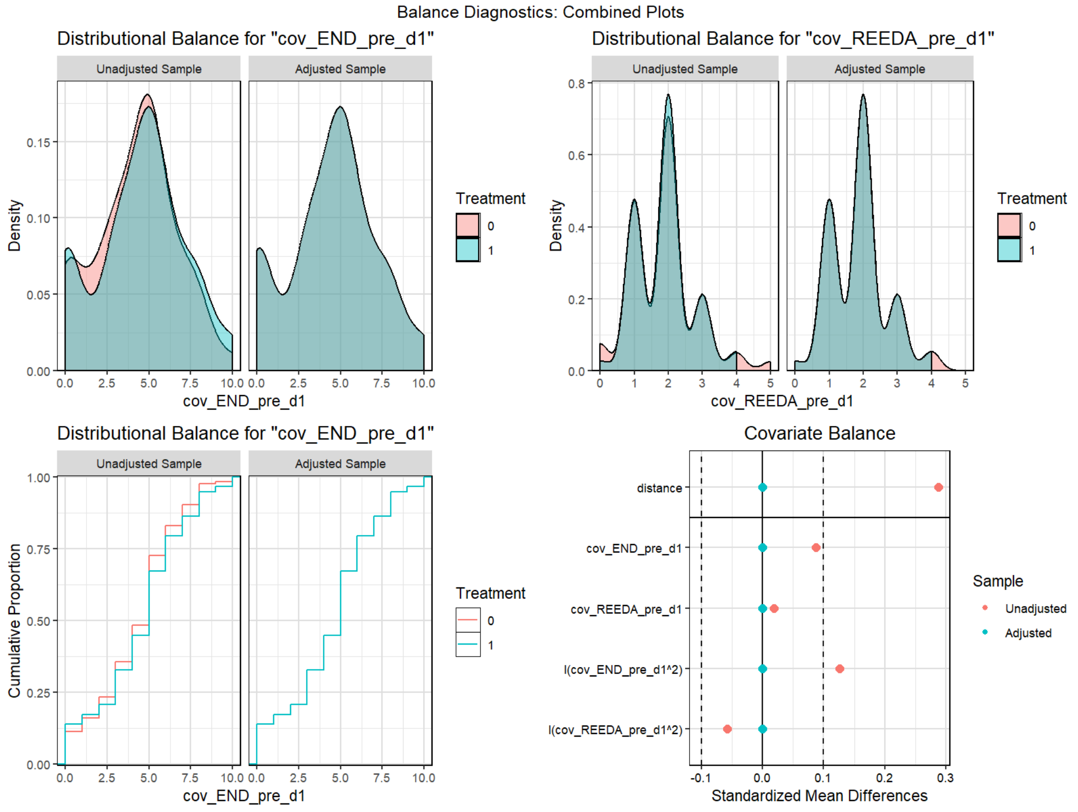

Others:

Examining the region of common support (*Graphical Analysis*)

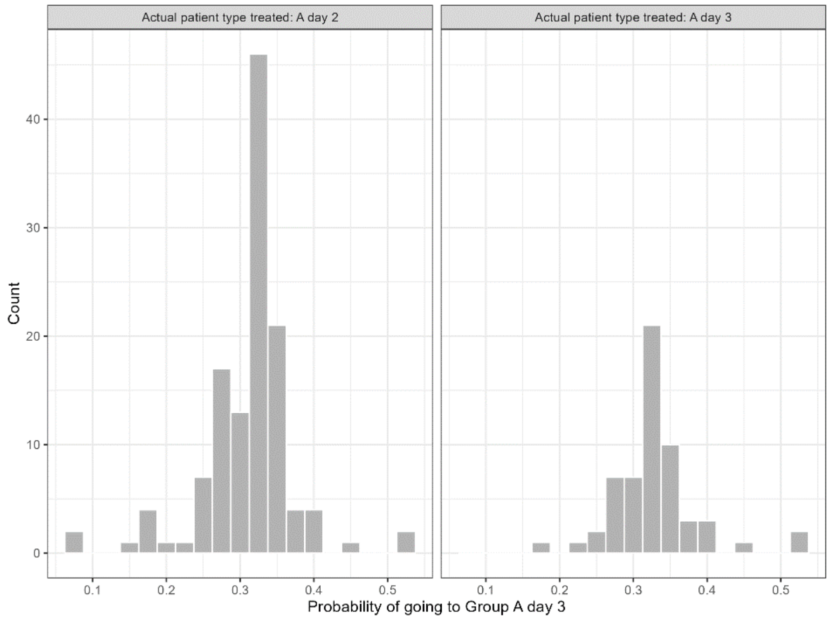

Estimate Treatment Effects

Using Welch Two Sample t-test

data: outcome_END by treatment

t = 1.6968, df = 113.41, **p-value = 0.09248 (alfa 10% sig.)**

alternative hypothesis: true difference in means between group 0 and group 1 is not equal to 0

95 percent confidence interval:

-0.1184565 1.5322496

sample estimates:

mean in group 0	mean in group 1
3.275862	2.568966

Using Ordinary Least Squares (OLS)

PAIN – END scale

Model Without Covariables

Model formula = outcome_END ~ treatment, data = mdata_ad3

Residuals:

Min	1Q	Median	3Q	Max
-3.2759	-1.7457	-0.2759	1.7241	5.4310

Coefficients:

	Estimate	Std. Error	t value	Pr(>|t|)
(Intercept)	3.2759	0.2946	11.120	<2e-16
treatment1	-0.7069	0.4166	-1.697	**0.0925**

Residual standard error: 2.244 on 114 degrees of freedom

Multiple R-squared: 0.02463, Adjusted R-squared: 0.01608

F-statistic: 2.879 on 1 and 114 DF, p-value: 0.09247

Model With Covariables

Model formula = outcome_END ~ treatment + cov_END_pre_d1 + cov_REEDA_pre_d1, data = mdata_ad3

Residuals:

Min	1Q	Median	3Q	Max
-4.1536	-1.5319	-0.2291	1.2620	5.1993

Coefficients:

	Estimate	Std. Error	t value	Pr(>|t|)
(Intercept)	0.5298	0.5264	1.007	0.31634
treatment1	-0.7069	0.3612	-1.957	**0.05282**
cov_END_pre_d1	0.3060	0.0721	4.244	4.55e-05

cov_REEDA_pre_d1	0.7409	0.2381	3.111	0.00236

Residual standard error: 1.945 on 112 degrees of freedom

Multiple R-squared: 0.2797, Adjusted R-squared: 0.2604

F-statistic: 14.5 on 3 and 112 DF, p-value: 4.805e-08

REEDA Scale

Model Without Covariables

Model formula = outcome_REEDA ~ treatment, data = mdata_ad3

Residuals:

Min	1Q	Median	3Q	Max
-1.5690	-0.5690	-0.1207	0.4310	2.4310

Coefficients:

	Estimate	Std. Error	t value	Pr(>|t|)
(Intercept)	1.5690	0.1002	15.659	<2e-16
treatment1	-0.4483	0.1417	-3.164	**0.002**

Residual standard error: 0.7631 on 114 degrees of freedom

Multiple R-squared: 0.08071, Adjusted R-squared: 0.07264

F-statistic: 10.01 on 1 and 114 DF, p-value: 0.001998

Model With Covariables

Model formula = outcome_REEDA ~ treatment + cov_END_pre_d1 + cov_REEDA_pre_d1, data = mdata_ad3

Residuals:

Min	1Q	Median	3Q	Max
-1.14891	-0.26087	0.04527	0.33883	1.50954

Coefficients:

	Estimate	Std. Error	t value	Pr(>|t|)
(Intercept)	0.15233	0.13019	1.170	0.244
treatment1	-0.44828	0.08933	-5.018	**1.98e-06**
cov_END_pre_d1	0.01599	0.01783	0.897	0.372
cov_REEDA_pre_d1	0.72243	0.05889	12.267	< 2e-16

Residual standard error: 0.481 on 112 degrees of freedom

Multiple R-squared: 0.6411, Adjusted R-squared: 0.6315

F-statistic: 66.68 on 3 and 112 DF, p-value: < 2.2e-16

Comparison: A d2 vs C

- Pre-analysis using non-matched data

• Difference-in-means: outcome variable

with(data_compare_b_c, t.test(outcome_END_pos ~ treatment))

Results:

Welch Two Sample t-test

data: outcome_END by treatment

t = 1.5147, df = 53.23, **p-value = 0.1358**

alternative hypothesis: true difference in means between group 1 and group 0 is not equal to 0

95 percent confidence interval:

-0.266604 1.911842

sample estimates:

mean in group 1 mean in group 0

2.114286 1.291667

Difference-in-means: pre-treatment covariates

[1]Welch Two Sample t-test

data: cov_END_pre by treatment

t = 2.1366, df = 52.361, **p-value = 0.03732**

alternative hypothesis: true difference in means between group 1 and group 0 is not equal to 0

95 percent confidence interval:

0.08386636 2.66613364

sample estimates:

mean in group 1 mean in group 0

3.000 1.625

[2] Welch Two Sample t-test

data: cov_REEDA_pre by treatment

t = 1.1885, df = 55.193, **p-value = 0.2397**

alternative hypothesis: true difference in means between group 1 and group 0 is not equal to 0

95 percent confidence interval:

-0.2009279 0.7866422

sample estimates:

mean in group 1 mean in group 0

1.542857 1.250000

sample estimates:

mean in group 0 mean in group 1

1.291667 2.967742

Pre-matching analysis

Table 4 Baseline characteristics of our data.

	1	0	p	SMD
n	35	24		
cov_END_pre (mean (SD))	3.00 (2.56)	1.62 (2.34)	0.040	0.562
cov_REEDA_pre (mean (SD))	1.54 (1.04)	1.25 (0.85)	0.257	0.309
outcome_END (mean (SD))	2.11 (2.19)	1.29 (1.94)	0.144	0.397
outcome_REEDA (mean (SD))	1.54 (1.04)	1.25 (0.74)	0.239	0.325

The numbers 0 and 1 in the first row indicate treatment groups: 0 means 'C' while 1 means 'B'. The variable n is the number of patients, cov_END_pre and cov_REEDA_pre are the independent variables, and outcome_END_pos is the indicator of pain scale post treatment. SMD, standardized mean difference; SD, [illegible]

Estimating Propensity Score (Ps)

After choosing the best model for PS Estimation

Model: non linear with GAM + full

Model formula:

matchit(treatment ~ cov_END_pre + cov_REEDA_pre + I(cov_END_pre^2) + I(cov_REEDA_pre^2) + cov_END_pre:cov_REEDA_pre, data = data_compare_b_c, method = "full", distance = "GAMlogit")

Graphical Analysis:

A Love plot compares the MEDs of covariates before and after matching, where two vertical dotted lines are drawn as the 0.1 MED threshold, and each point represents the MED of a covariate before or after PSM adjustment. If a point falls between two lines, the matching covariate has been balanced.

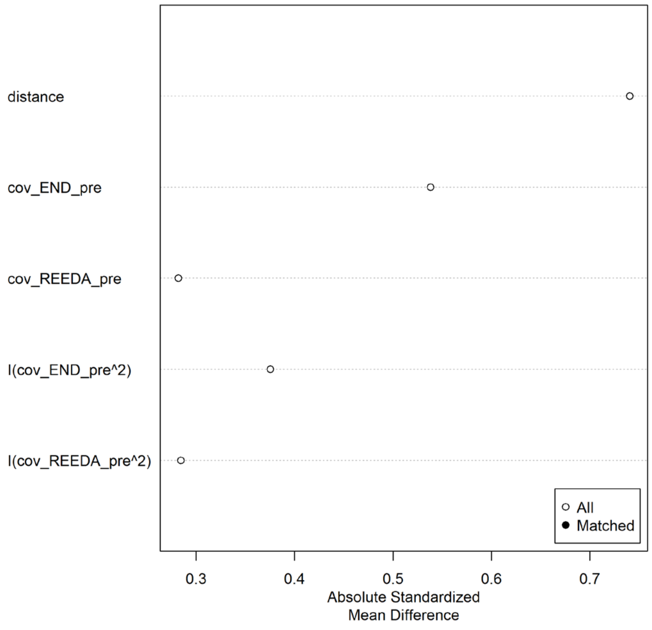

Others

Plot: Visualization of distribution of covariates before and after propensity score matching

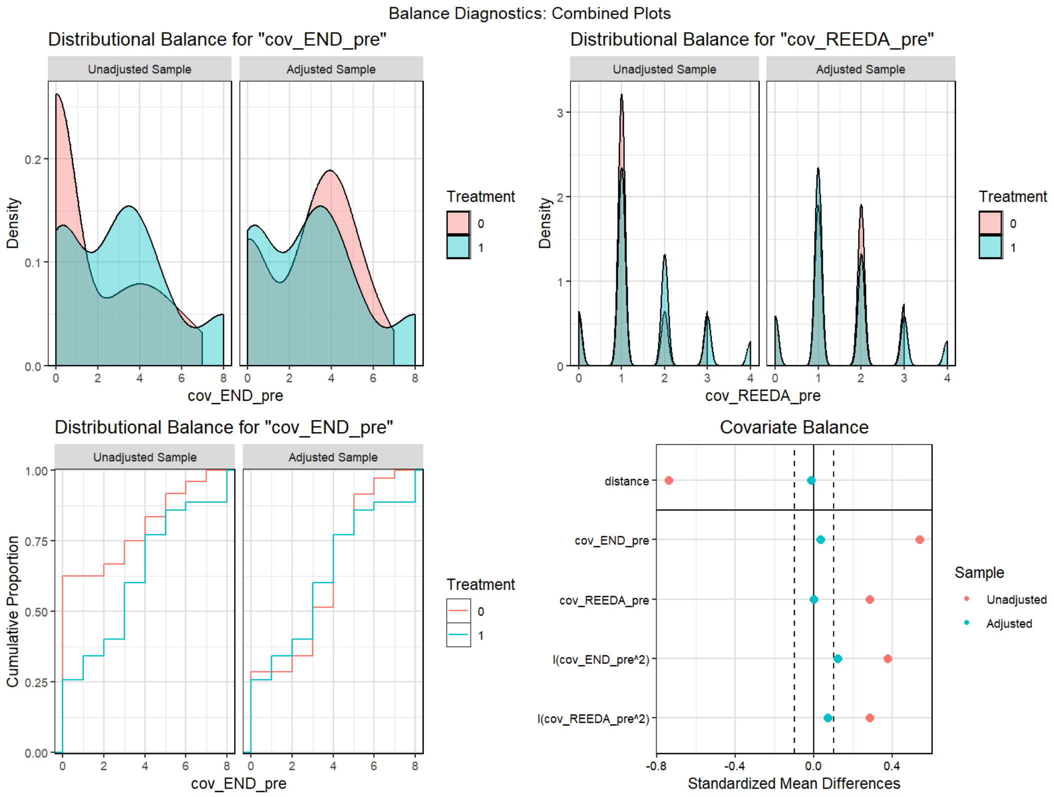

Examining the region of common support (*Graphical Analysis*)

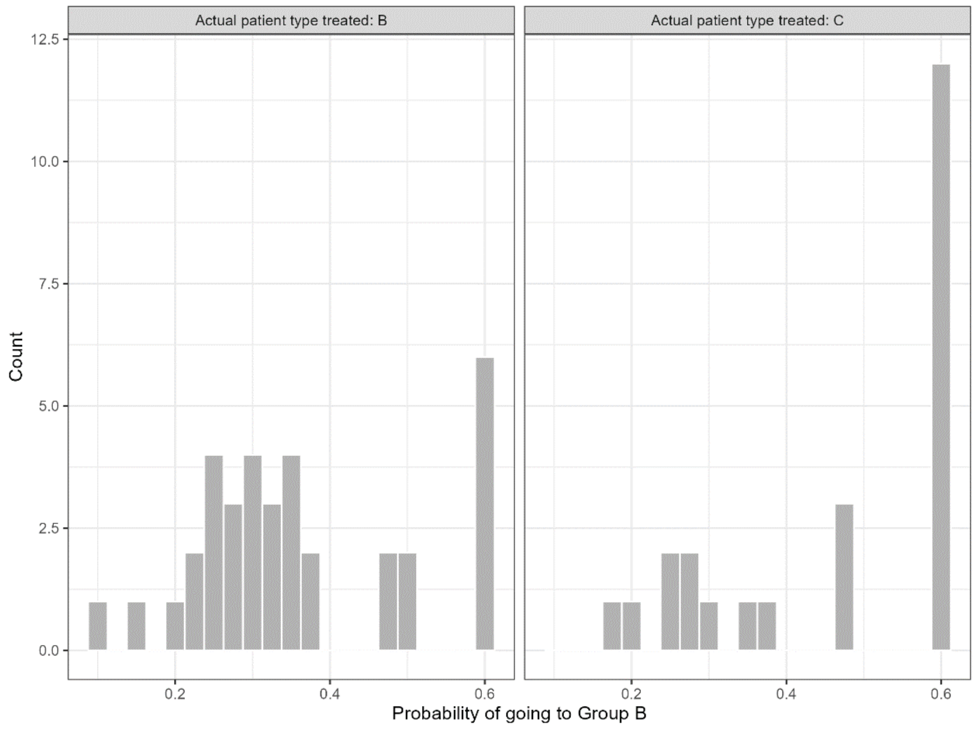

Estimate Treatment Effects

Using Welch Two Sample t-test

data: outcome_END by treatment

t = 1.5147, df = 53.23, **p-value = 0.1358**

alternative hypothesis: true difference in means between group 1 and group 0 is not equal to 0

95 percent confidence interval:

-0.266604 1.911842

sample estimates:

mean in group 1 mean in group 0

2.114286   1.291667

Using Ordinary Least Squares (OLS)

PAIN – END Scale

Model Without Covariables

z5$zelig(formula = outcome_END ~ treatment, data = mdata_2)

Residuals:

Min	1Q	Median	3Q	Max
-2.114	-1.292	-1.114	1.708	5.886

Coefficients:

	Estimate	Std. Error	t value	Pr(>|t|)
(Intercept)	2.1143	0.3544	5.967	1.63e-07
treatment0	-0.8226	0.5556	-1.481	**0.144**

Residual standard error: 2.096 on 57 degrees of freedom

Multiple R-squared: 0.03704,   Adjusted R-squared: 0.02014

F-statistic: 2.192 on 1 and 57 DF, p-value: 0.1442

Model With Covariables

z5$zelig(formula = outcome_END ~ treatment + cov_END_pre + cov_REEDA_pre,
    data = mdata_2)

Residuals:

Min	1Q	Median	3Q	Max
-3.4999	-0.1776	-0.0522	0.4776	3.8293

Coefficients:

	Estimate	Std. Error	t value	Pr(>|t|)
(Intercept)	0.052197	0.341827	0.153	0.879
treatment0	0.125366	0.342672	0.366	**0.716**
cov_END_pre	0.690915	0.082751	8.349	2.36e-11

cov_REEDA_pre	-0.006907	0.211560	-0.033	0.974

Residual standard error: 1.246 on 55 degrees of freedom

Multiple R-squared: 0.672, Adjusted R-squared: 0.6541

F-statistic: 37.56 on 3 and 55 DF, p-value: 2.407e-13

REEDA Scale

Model Without Covariables

z5$zelig(formula = outcome_REEDA ~ treatment, data = mdata_2)

Residuals:

Min	1Q	Median	3Q	Max
-1.5429	-0.5429	-0.2500	0.4571	2.4571

Coefficients:

	Estimate	Std. Error	t value	Pr(>|t|)
(Intercept)	1.5429	0.1570	9.826	7.13e-14
treatment0	-0.2929	0.2462	-1.190	**0.239**

Residual standard error: 0.9289 on 57 degrees of freedom

Multiple R-squared: 0.02422, Adjusted R-squared: 0.007105

F-statistic: 1.415 on 1 and 57 DF, p-value: 0.2391

Model With Covariables

z5$zelig(formula = outcome_REEDA ~ treatment + cov_END_pre +
cov_REEDA_pre, data = mdata_2)

Residuals:

Min	1Q	Median	3Q	Max
-1.00208	-0.05180	-0.00208	0.04168	0.90249

Coefficients:

	Estimate	Std. Error	t value	Pr(>|t|)
(Intercept)	0.107031	0.070783	1.512	0.136
treatment0	-0.009522	0.070958	-0.134	**0.894**
cov_END_pre	0.013399	0.017136	0.782	0.438
cov_REEDA_pre	0.904574	0.043808	20.648	<2e-16

Residual standard error: 0.2579 on 55 degrees of freedom

Multiple R-squared: 0.9274, Adjusted R-squared: 0.9235

F-statistic: 234.3 on 3 and 55 DF, p-value: < 2.2e-16
