# Supplementary material for: Effects of Photobiomodulation Therapy on Pain and Healing of Episiotomies and Grade 2 and 3 Perineal Lacerations After Vaginal Delivery: A Prospective Observational Cohort Study
Source: Med Sci (Basel). 2026 Mar 6;14(1):125. doi: 10.3390/medsci14010125 (PMC13027586; doi:10.3390/medsci14010125)
Supplement: Supplementary file 1 [file medsci-14-00125-s001.zip › Table S1.pdf]

Table S1. Comparison of pain scores (NPS) between participants with one laser sessions and those without laser after PSM test.

NPS				
	Estimate	Std. Error	t value	Pr (>|t|)
(Intercept)	2.1143	0.3544	5.967	1.63e-07
treatment0	-0.8226	0.5556	-1.481	**0.144**

Residual standard error: 2.096 on 57 degrees of freedom

Multiple R-squared: 0.03704, Adjusted R-squared: 0.02014

F-statistic: 2.192 on 1 and 57 DF, p-value: 0.1442
