# Supplementary material for: Effects of Photobiomodulation Therapy on Pain and Healing of Episiotomies and Grade 2 and 3 Perineal Lacerations After Vaginal Delivery: A Prospective Observational Cohort Study
Source: Med Sci (Basel). 2026 Mar 6;14(1):125. doi: 10.3390/medsci14010125 (PMC13027586; doi:10.3390/medsci14010125)
Supplement: Supplementary file 1 [file medsci-14-00125-s001.zip › Table S2.pdf]

Table S2. Comparison of pain scores (NPS) between participants with two laser sessions and those with one laser after PSM test.

NPS				
	Estimate	Std. Error	t value	Pr (>|t|)
(Intercept)	0.45848	0.33441	1.371	0.175
treatment1	-0.31228	0.30241	-1.033	**0.306**

Residual standard error: 1.263 on 66 degrees of freedom

Multiple R-squared: 0.6304, Adjusted R-squared: 0.6136

F-statistic: 37.53 on 3 and 66 DF, p-value: 2.848e-14
