# Supplementary material for: Effects of Photobiomodulation Therapy on Pain and Healing of Episiotomies and Grade 2 and 3 Perineal Lacerations After Vaginal Delivery: A Prospective Observational Cohort Study
Source: Med Sci (Basel). 2026 Mar 6;14(1):125. doi: 10.3390/medsci14010125 (PMC13027586; doi:10.3390/medsci14010125)
Supplement: Supplementary file 1 [file medsci-14-00125-s001.zip › Table S3.pdf]

Table S3: Inter-subgroup comparison of REEDA scores according to the number of laser sessions. REEDA scores across subgroups receiving 2, 1, or 0 laser sessions

	REEDA Day 1		REEDA Day 2		REEDA Day 3		
	**Mean**	**S.D.**	**Mean**	**S.D.**	**Mean**	**S.D.**	**p**
Subgroup 0 session (n 12)	1.42	1	1.5	0.90	1.5	1.09	0.01
IC 95%	0.85-1.98		0.98-2.01		0.88-2.11		
Subgroup 1 session (n 17)	1.29	0.85	0.94	0.97	0.88	0.86	0.08
IC 95%	0.89-1.70		0.48-1.4		0.47-1.29		
Subgroup 2 session (n 73)	1.92	0.80	1.63	0.83	1.12	0.69	**<0.0001***
IC 95%	1.72.10		1.44-1.82		0.96-1.28		

Repeated ANOVA measures. *: significantly different $p < 0.05$.
