# Supplementary material for: Effects of Photobiomodulation Therapy on Pain and Healing of Episiotomies and Grade 2 and 3 Perineal Lacerations After Vaginal Delivery: A Prospective Observational Cohort Study
Source: Med Sci (Basel). 2026 Mar 6;14(1):125. doi: 10.3390/medsci14010125 (PMC13027586; doi:10.3390/medsci14010125)
Supplement: Supplementary file 1 [file medsci-14-00125-s001.zip › Table S4.pdf]

Table S4. Comparison of REEDA Scores between participants with two laser sessions and those without laser after PSM test.

**Coefficients:**				
	**Estimate**	**Std. Error**	**t value**	**Pr(>|t|)**
(Intercept)	0.322124	0.110201	2.923	0.00403
treatment1	-0.203808	0.114048	-1.787	**0.07604**

Residual standard error: 0.4708 on 144 degrees of freedom

Multiple R-squared: 0.6891, Adjusted R-squared: 0.6827

F-statistic: 106.4 on 3 and 144 DF, p-value: < 2.2e-16
