# Supplementary material for: Effects of Photobiomodulation Therapy on Pain and Healing of Episiotomies and Grade 2 and 3 Perineal Lacerations After Vaginal Delivery: A Prospective Observational Cohort Study
Source: Med Sci (Basel). 2026 Mar 6;14(1):125. doi: 10.3390/medsci14010125 (PMC13027586; doi:10.3390/medsci14010125)
Supplement: Supplementary file 1 [file medsci-14-00125-s001.zip › Table S5.pdf]

Table S5: Comparison of REEDA scores between participants with one laser sessions and those without laser after PSM test.

Between the 1 session and 0 session subgroups coefficients				
	Estimate	Std. Error	t value	Pr (>|t|)
(Intercept)	1.5429	0.1570	9.826	7.13e-14
treatment0	-0.2929	0.2462	-1.190	**0.239**

Residual standard error: 0.9289 on 57 degrees of freedom
Multiple R-squared: 0.02422, Adjusted R-squared: 0.007105
F-statistic: 1.415 on 1 and 57 DF, p-value: 0.2391
