# Supplementary material for: Effects of Photobiomodulation Therapy on Pain and Healing of Episiotomies and Grade 2 and 3 Perineal Lacerations After Vaginal Delivery: A Prospective Observational Cohort Study
Source: Med Sci (Basel). 2026 Mar 6;14(1):125. doi: 10.3390/medsci14010125 (PMC13027586; doi:10.3390/medsci14010125)
Supplement: Supplementary file 1 [file medsci-14-00125-s001.zip › Table S6.pdf]

Table S6. Comparison of REEDA scores between participants with one laser sessions and those with two laser after PSM test.

Between the 2-session and 1-session subgroups coefficients				
	Estimate	Std. Error	t value	Pr (>|t|)
(Intercept)	1.657e-16	4.735e-17	3.499e+00	0.000841
treatment1	-1.147e-17	4.282e-17	-2.680e-01	**0.789559**

Residual standard error: 1.788e-16 on 66 degrees of freedom

Multiple R-squared: 1, Adjusted R-squared: 1

F-statistic: 5.925e+32 on 3 and 66 DF, p-value: < 2.2e-16
