# Supplementary figures and images for: Effects of Photobiomodulation Therapy on Pain and Healing of Episiotomies and Grade 2 and 3 Perineal Lacerations After Vaginal Delivery: A Prospective Observational Cohort Study
Source: Med Sci (Basel). 2026 Mar 6;14(1):125. doi: 10.3390/medsci14010125 (PMC13027586; doi:10.3390/medsci14010125)

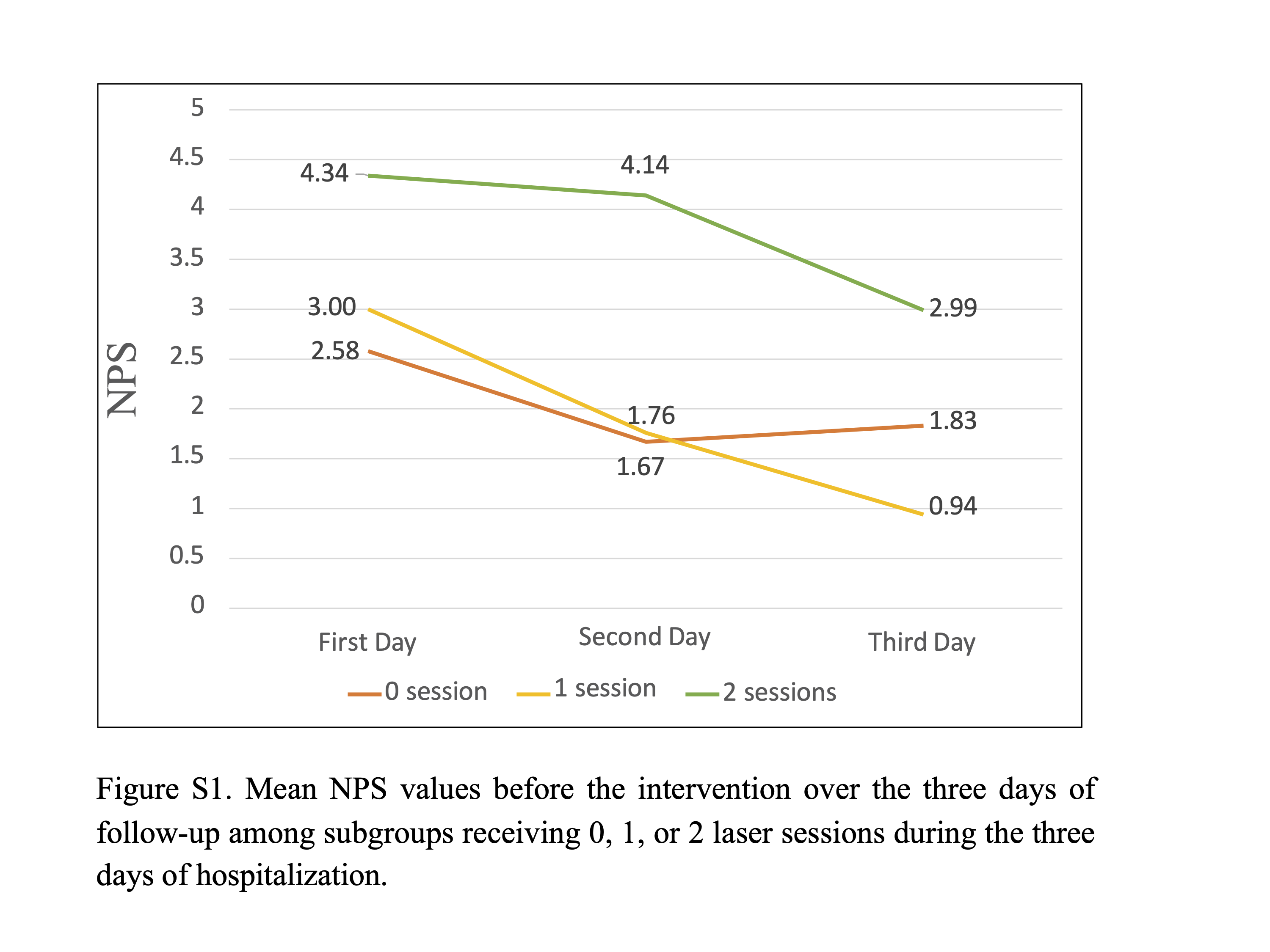

Supplement: Supplementary file 1 [file medsci-14-00125-s001.zip › Figure S1.png]

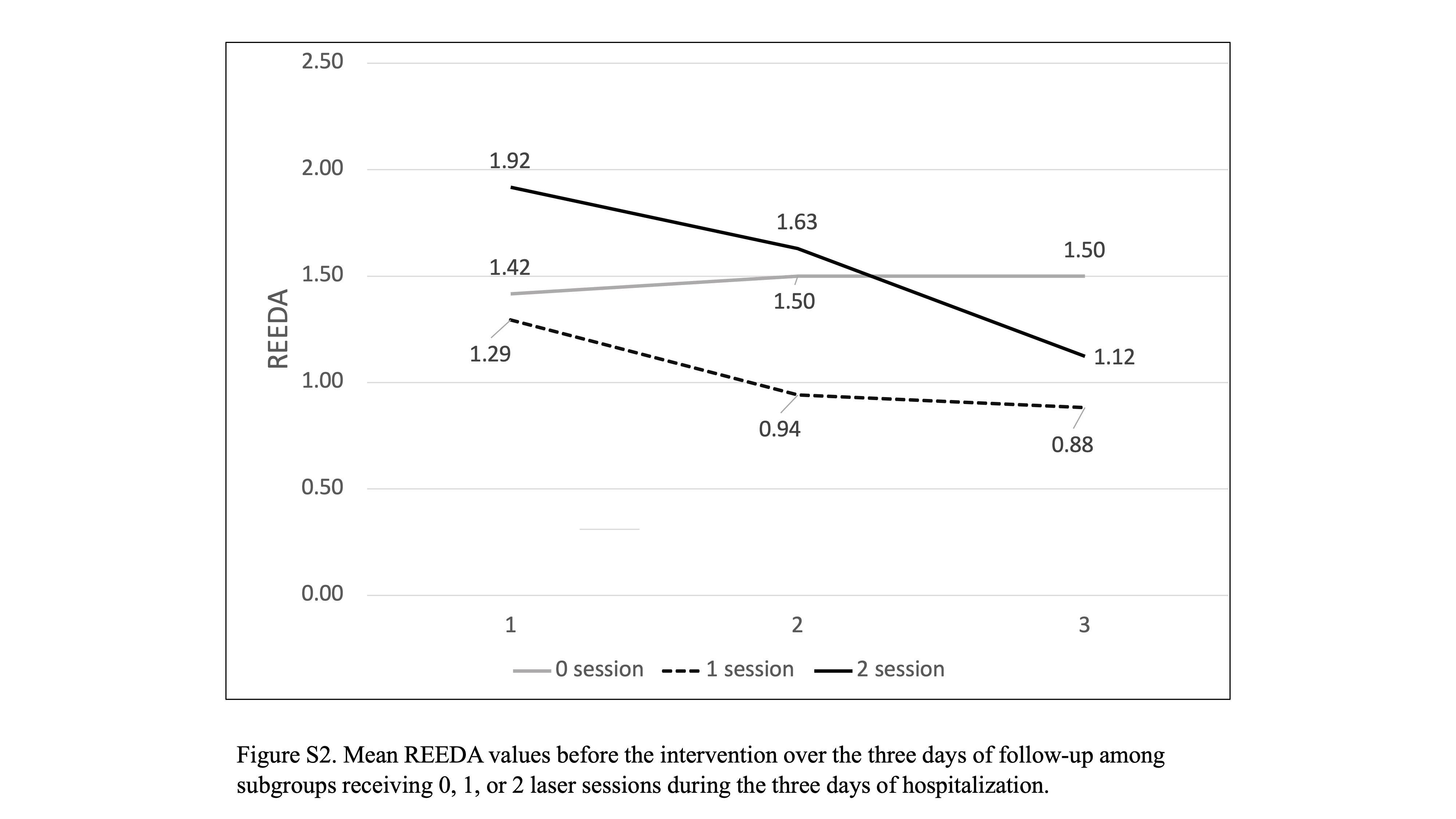

Supplement: Supplementary file 1 [file medsci-14-00125-s001.zip › Figure S2.png]
